# Special Types of Breast Cancer: Clinical Behavior and Radiological Appearance

**DOI:** 10.3390/jimaging10080182

**Published:** 2024-07-29

**Authors:** Marco Conti, Francesca Morciano, Silvia Amodeo, Elisabetta Gori, Giovanna Romanucci, Paolo Belli, Oscar Tommasini, Francesca Fornasa, Rossella Rella

**Affiliations:** 1 UOC di Radiologia Toracica e Cardiovascolare, Dipartimento di Diagnostica per Immagini e Radioterapia Oncologica, Fondazione Policlinico Universitario Agostino Gemelli IRCCS, Largo A. Gemelli 8, 00168 Rome, Italy; marco.conti@policlinicogemelli.it (M.C.); paolo.belli@policlinicogemelli.it (P.B.); 2 Facoltà di Medicina e Chirurgia, Università Cattolica Sacro Cuore, Largo F. Vito 1, 00168 Rome, Italy; francesca.morciano@hotmail.com (F.M.); silvia.amodeo02@icatt.it (S.A.); elisabetta.gori01@icatt.it (E.G.); 3 UOSD Breast Unit ULSS9, Ospedale di Marzana, Piazzale Lambranzi 1, 37142 Verona, Italy; francesca.fornasa@aulss9.veneto.it; 4 UOC Diagnostica per Immagini, Dipartimento Emergenza e Accettazione, Ospedale G.B. Grassi, Via Gian Carlo Passeroni, 28, 00122 Rome, Italy; oscar.tommasini@aslroma3.it (O.T.); rossella.rella@aslroma3.it (R.R.)

**Keywords:** breast imaging, breast cancer, special types, invasive lobular carcinoma, conventional and advanced imaging

## Abstract

Breast cancer is a complex disease that includes entities with different characteristics, behaviors, and responses to treatment. Breast cancers are categorized into subgroups based on histological type and grade, and these subgroups affect clinical presentation and oncological outcomes. The subgroup of “special types” encompasses all those breast cancers with insufficient features to belong to the subgroup “invasive ductal carcinoma not otherwise specified”. These cancers account for around 25% of all cases, some of them having a relatively good prognosis despite high histological grade. The purpose of this paper is to review and illustrate the radiological appearance of each special type, highlighting insights and pitfalls to guide breast radiologists in their routine work.

## 1. Introduction

Breast cancer (BC) encompasses a set of heterogeneous entities characterized by histological and biological peculiarities, each one with its own clinical behavior and response to treatment.

BC is classified into several subgroups according to histological grade [1] and type [2]. In particular, the latter refers to the growth pattern of cancerous cells: the importance of this pathological difference is reflected in the distinctive clinical presentations and oncological outcomes of the different subtypes.

Most BCs (75–80%) are part of the so-called “invasive carcinoma of no special type (NST)” subgroup [3], a subgroup that encompasses all those entities that do not manifest sufficient features to be classified in one of the “special types” subgroups. NST breast carcinoma, previously known as invasive ductal carcinoma not otherwise specified (IDC-NOS), accounts for 70–80% of all invasive breast carcinomas and is the most common histotype in both men and women. It is characterized by remarkable variety in terms of morphology of tumoral cells and by the presence of tubular or glandular structures, as well as by an absence of growth patterns or cytological characteristics typical of special histotypes [4].

The World Health Organization (WHO) Classification of Tumors of the Breast is considered the gold standard for the diagnosis of BCs, providing the international standards for their classification. The 5th and latest edition was published in 2019; in it, several special types were recognized, representing together up to 25% of all classifications [5].

The knowledge and the recognition of special types are crucial for clinical management, as each is unique in its response to therapy and outcomes.

The aim of this review is to describe the clinical behavior and to illustrate the radiological appearance of most special BC types, highlighting insights and pitfalls to guide radiologists in their routine work.

In recent years, especially after the COVID-19 pandemic highlighted the need for large-scale early diagnosis, many studies have shown that the use of artificial intelligence applied to radiological images is a valuable resource, but it is critical that computational models achieve adequate diagnostic accuracy and become reliable and repeatable. For this reason, further studies are desirable before such models are included in clinical practice [6].

AI algorithms have been broadly applied to BC diagnosis and personalized medicine, especially in the detection of BC and lymph-node metastasis, and in biomarker quantification, prediction of genetic mutations, and prediction of clinical outcomes [7].

Despite the increasing role of artificial intelligence as a valuable tool in BC detection, there are still many factors that influence software processing abnormality scores, with unsatisfactory performance under many conditions, such as dense breasts, small lesions, isodense lesions, and asymmetries [8]. Great progress is being made in the development of new AI systems that can be applied to multimodal imaging for image analysis. This progress is intended to support the work of clinicians through valuable intelligent agents [9,10]; however, it is clear that the radiologist plays a pivotal role in the diagnosis of BC and needs to know both the clinical and multimodal-imaging features of each BC subtype.

In this work, we deal with most of the special subtypes, excluding only some extremely rare types that are not specifically listed in the next paragraphs and that have not been treated extensively because of their extreme rarity [11].

All images provided in this work are original and were extracted from an internal database of the centers involved in the study, specifically Policlinico Universitario A. Gemelli IRCCS, Hospital of Marzana, and G.B. Grassi Hospital. Written informed consent to publish this paper has been obtained from the patients.

## 2. Lobular (Classic, Pleomorphic and Florid Types)

Invasive lobular carcinoma (ILC) is the most common special type of BC, representing up to 15% of all BCs [12], with a higher incidence in peri-menopausal women (40–49 years) [13]. ILC is associated with a poorer long-term prognosis than IDC [14]. The response to chemotherapy is often poor [15].

Most ILCs express estrogen receptors (ERs) and progesterone receptors (PRs), while human epidermal growth factor receptor-2 (HER2) is usually not expressed [12].

Microscopically, this type is characterized by loss of cellular adhesion and a resultant discohesive growth pattern. The classic form of ILC, the most common one, is distinguished from other morphological variants, such as the pleomorphic, solid, alveolar, and tubulo-lobular forms that typically coexist with it [14]. Each of them has a different clinical behavior, i.e., the solid and the pleomorphic ones are linked to a poor prognosis, whereas the tubulo-lobular and alveolar ones are associated with a good prognosis [16].

ILC tends to be more frequently multifocal or multicentric than does IDC [17].

The available data on the status of the axillary lymph nodes in ILC compared to IDC are conflicting, although the majority of published studies found no significant difference in nodal involvement between these two histopathological subtypes [18]. Furthermore, metastatic spread is found in two thirds of cases at diagnosis; compared to IDC, ILC has a higher propensity to spread to the peritoneum or retroperitoneum, urogenital and gastrointestinal tracts, leptomeninges, and myocardium; however, the rate of metastasis to the liver, pleura, and bone is similar between the two groups [17].

ILC generally presents as a palpable mass, while in mammography (MX), it is frequently overlooked, with a reported false-negative rate of 19–43%; this can be explained by its diffuse infiltrative growth pattern [14].

In particular, MX has 57–71% sensitivity in detecting ILC [19], which is even lower (down to 11%) in dense breasts [20]. On MX, ILC presents as a mass with spiculated/irregular or ill-defined margins in most cases because of its infiltrative growth pattern, or it presents as a focal asymmetry [19,21], which is usually seen on cranio-caudal projection [17] with a radiopacity similar to that of normal breast parenchyma, which makes its diagnosis challenging [21].

In 14–25% of cases, it appears as an architectural distortion; for this reason, digital breast tomosynthesis (DBT) plays an important role in its detection [22]. DBT increases ILC’s conspicuity by clearly depicting architectural distortion and improving the early diagnosis and detection of this type of cancer [23,24]. Onega et al. described a higher detection rate for ILC with DBT vs. MX, which further increased in women with dense breasts [22]. A well-circumscribed round or oval-shaped mass is rarely observed (1% of cases) when the neoplasm develops a stromal reaction; likewise, microcalcifications are present only in 1–25% of tumors [19]. Additionally, ILC has never been described as presenting with microcalcifications without other abnormalities; on the contrary, IDC presents with microcalcifications in about 16.7% of cases [25].

The detection of this subtype improves with ultrasound (US), which has a sensitivity of 68–98% [21].

**Figure 1:**
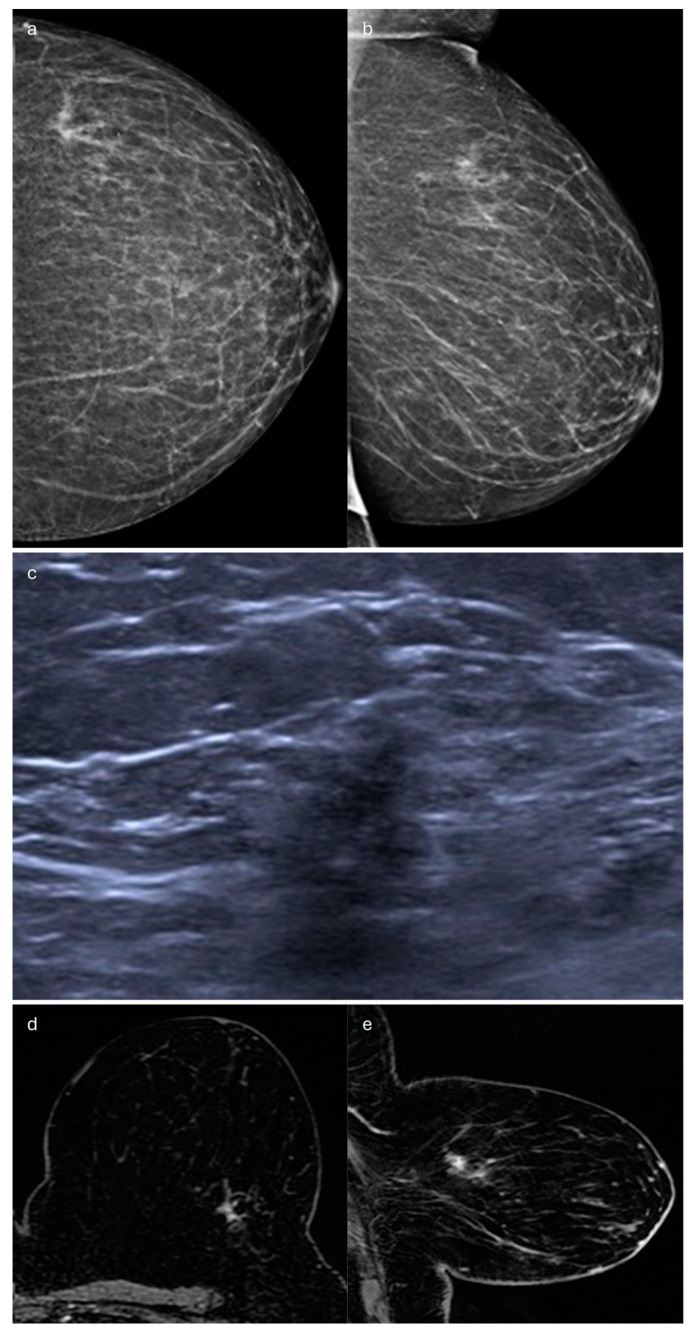
Invasive lobular carcinoma in a 53-year-old patient. (**a**) Cranio-caudal and (**b**) medio-lateral oblique mammograms of the left breast show an architectural distortion in the upper-outer quadrant, (**c**) corresponding to an irregular-shaped hypoechoic lesion with spiculated margins on ultrasound. (**d**) Axial and (**e**) sagittal T1-weighted fat-suppressed dynamic contrast-enhanced MRI images reveal an irregular mass with spiculated margins in the upper-outer quadrant.

In most cases (60%), ILC sonographically appears as a hypoechoic irregular mass with posterior acoustic shadowing [17,19] (Figure 1). Isoechogenicity of the mass has also been described [26]. Kim et al. found that posterior acoustic shadowing was more specific to ILC than to IDC [25]. A mass without shadowing is observed in about 20% of cases, and shadowing without a distinct mass may also be seen [19].

Only 2–12% of ILCs appear on US as smooth, lobulated, or well-circumscribed masses [19]. Most masses show parallel orientation and vascularity, which is more frequently peripheral than internal [13]. Finally, a diffuse infiltrative inhomogeneous hypoechoic pattern with indistinct margins and no shadowing is frequently observed in ILC [21,27]. Wen et al. also described different US presentations of ILC subtypes: the HER2 subtype is significantly characterized by microlobulated margins, whereas the luminal A and luminal B subtypes are significantly characterized by spiculated margins and the basal-like subtype tends to have indistinct margins [13]. However, it is important to underline that 10% of ILCs still remain undetectable with this technique [19].

Magnetic resonance imaging (MRI) is the most accurate imaging modality to assess the local extent of ILC [28] and also represents a fundamental tool for identifying multifocality and multicentricity [14], with an overall sensitivity of 93–95%. For this reason, when ILC is histologically proven, a staging MRI before surgery is indicated [29]. The most common finding of ILC on MRI (up to 95%) is an irregular mass with spiculated or irregular margins (Figure 1). Less frequently, a non-mass enhancement (NME) is seen (in up to 69% of cases) [17]. Ductal, segmental, regional, or diffuse distribution patterns of NME can be found, given the extremely variable appearance of this special type on MRI [18]. Multiple enhancing foci around the main lesion, associated with enhancing septa, strands, and glandular distortion, represent another less common MRI finding [21]. In a cohort of 162 patients with ILC who underwent MRI, Bartosz Dołęga-Kozierowski et al. found 113 mass lesions (about 70%) and 49 NMEs [28]. Both mass and non-mass lesions typically have a restricted diffusion and heterogeneous contrast enhancement. With regard to kinetic curves, their evaluation is not so useful because although most lesions have a type-II or -III dynamic curve, a type-I enhancement curve is possible, so kinetic curves do not provide discrimination between ILC and a benign finding [28]. It should also be considered that kinetic curves can be misleading when applied to NME, since malignant tumors that show NME may exhibit typically benign curves, as they do not have strong angiogenic activity [30,31].

**Figure 2:**
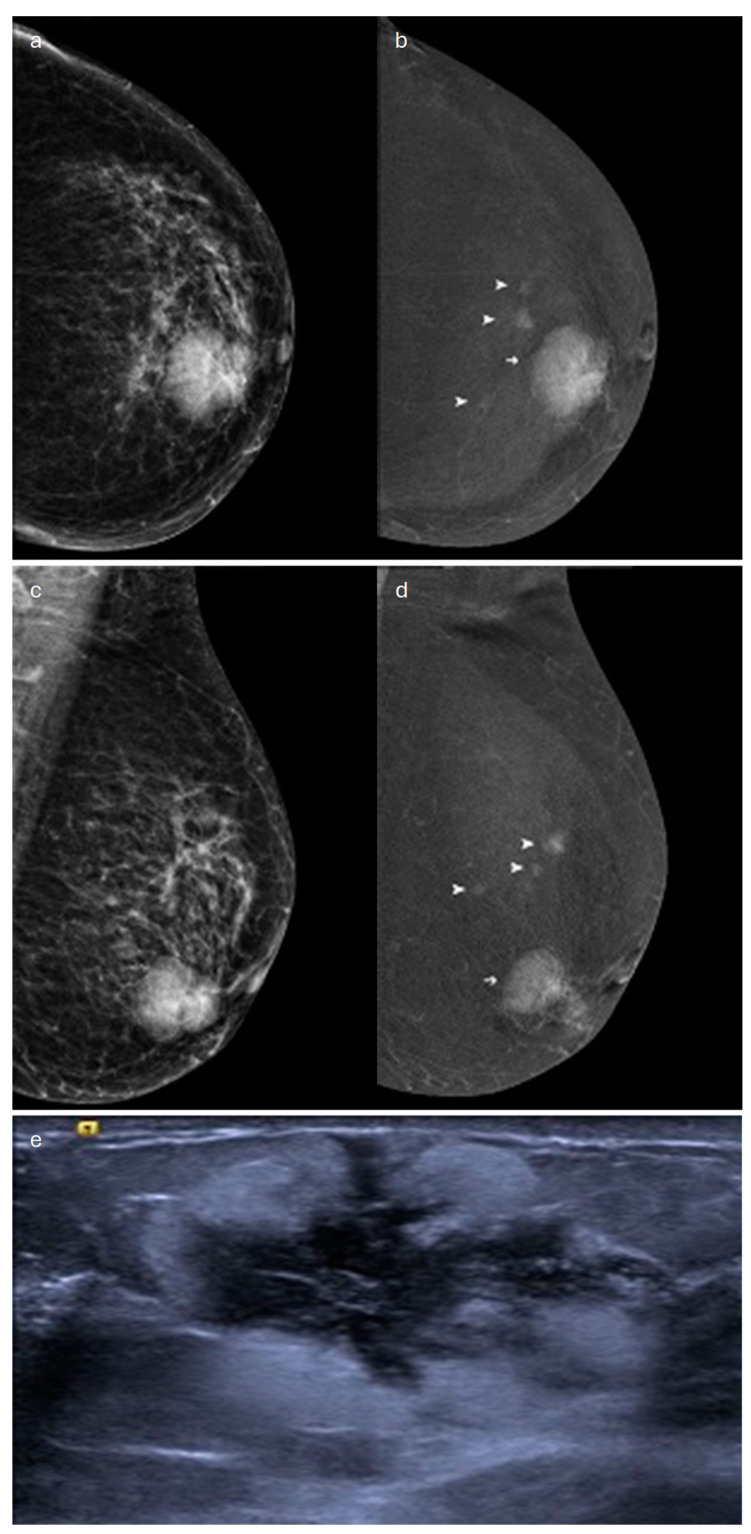
Invasive lobular carcinoma in a 78-year-old patient. Contrast-enhancement mammography: (**a**) low-energy cranio-caudal and (**c**) medio-lateral oblique images of the left breast show a high-density irregular mass, (**b**,**d**) corresponding to an area of mass enhancement on the recombined images (white arrow). Recombined images highlight the presence of three satellite nodules (white arrowheads), demonstrating the multicentricity of the disease. (**e**) US image shows an irregular hypoechoic mass with spiculated margins corresponding to the primary lesion, indicated by the white arrow in (**b**,**d**).

In terms of its appearance in Contrast-Enhanced Digital Mammography (CEDM), ILC mostly presents as an oval-shaped or irregular mass lesion with irregular margins and heterogeneous internal enhancement (Figure 2); in a minority of cases, it can be associated with NME or appears as an NME only (focal, regional or segmental). The observed kinetic curves are predominantly plateau-shaped [32].

Some authors have compared the diagnostic accuracy of CEDM with that of MX: the first technique was more accurate for the detection of ILC, the assessment of the extent of the disease, and the measurement of tumor size, highlighting its potential role both in the identification of ILC in symptomatic women overlooked by MX, US, and DBT and in loco-regional preoperative staging of this subtype [32,33].

In a recent study comparing the accuracy of CEDM to that of MRI in detecting multifocal and/or contralateral tumoral foci in preoperative staging of ILC, Lobbes et al. found that the latter has superior sensitivity but lower specificity than CEDM. Nonetheless, no significant differences were found in terms of diagnostic performance, measured as diagnostic odds ratio, between these two techniques [34].

In conclusion, ILC has a higher probability of presenting as multicentric or multifocal disease with nodal and distant metastases than does IDC-NST: this means that it is more likely to be diagnosed at a later stage, even though it tends to be lower-grade than IDC. Moreover, when ILC and IDC are compared at the same stage, the first shows worse long-term outcomes; hence, further studies and randomized clinical trials are needed to improve its detection, to test new neoadjuvant and endocrine therapies, and to develop a targeted treatment for ILC, now recognizable as a distinct pathology [22].

## 3. Tubular

Tubular cancer (TC) is a rare subtype of invasive BC with a reported incidence of about 1–5% [35,36,37,38]. It can present as a “pure” or a “mixed” type, the latter when in association with other types of cancer, in particular with ductal carcinoma in situ (DCIS) and IDC, and is associated with excellent survival rates in both cases [39].

Lymph-node metastases are rare [28]. However, some authors suggest that the lymph nodes can be involved even in small lesions (<10 mm); axillary staging is therefore always recommended [40].

**Figure 3:**
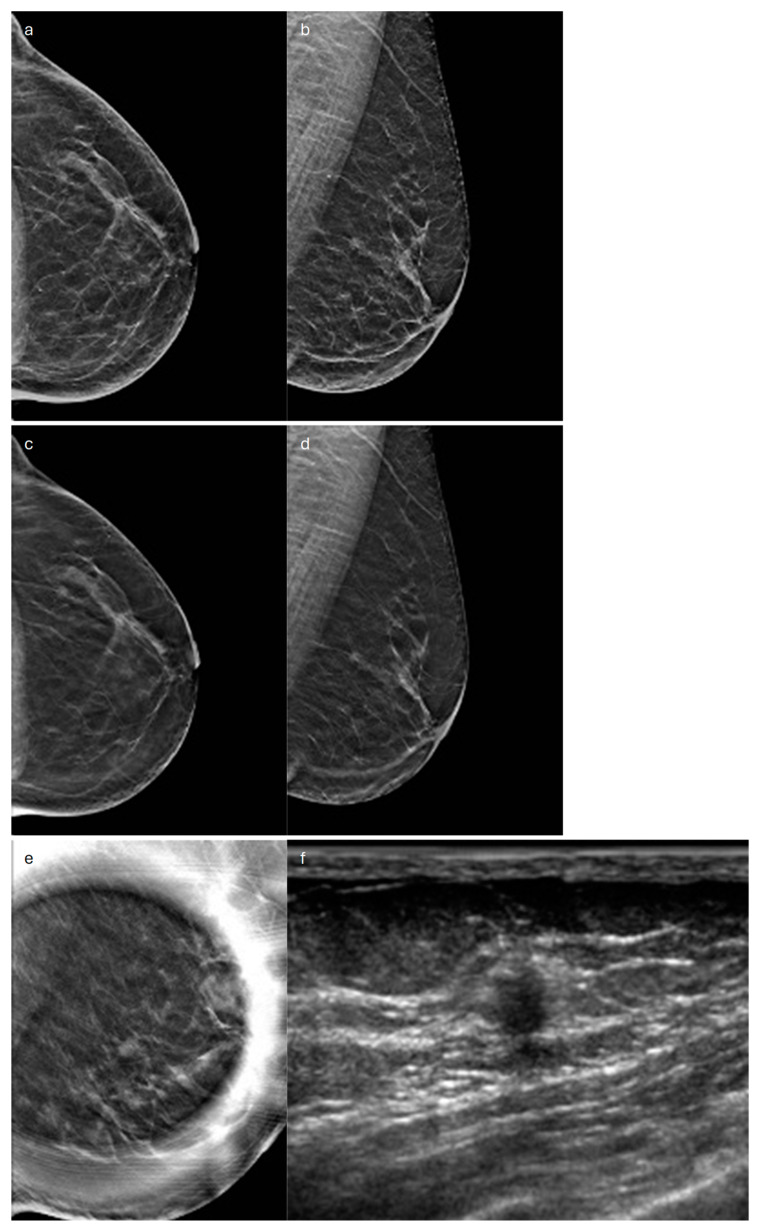
Tubular carcinoma. (**a**) Cranio-caudal and (**b**) medio-lateral oblique 2D synthetic mammograms (**c**), cranio-caudal and (**d**) medio-lateral oblique DBT images, and (**e**) spot compression view of the left breast showing a small opacity (**f**) corresponding to a hypoechoic mass with non-circumscribed margins on ultrasound.

In terms of its radiological appearance, on MX, TC usually manifests as an irregular mass with spiculated margins [41,42]. Also, on US, it cannot be distinguished from IDC-NST because it appears as a hypoechoic solid mass with non-circumscribed margins and posterior acoustic shadowing [41,42] (Figure 3).

DBT visualizes TC as a mass with long spicules or as an architectural distortion and, compared to MX, improves detection of this type of cancer [43] (Figure 3).

There are few reports in the literature describing the MRI characteristics of TCs. Linda et al. described them as irregularly shaped masses with non-circumscribed margins that are characterized by low-to-intermediate signal on T2-weighted sequences with heterogeneous enhancement, which results in a persistent kinetic curve [44]. However, in some cases, TC can manifest as a non-enhancing lesion, as reported by Ghai et al. [45].

In a limited number of patients, TCs may present as high-signal-intensity lesions with a characteristic hypointense internal septation-like appearance on T2-weighted sequences and low ADC values (0.85 ± 0.16 × 10^−3^ mm^2^/s); for the authors, these features may help in differentiating TCs from other BC subtypes [46].

BC special types do not show significant differences in shear wave elastography (SWE) characteristics, but among them, TCs are an exception: they have a stiffness for Emean and Emax similar to that of the benign range, although this can be explained in large part by their small size at diagnosis [47].

## 4. Mucinous

Mucinous cancer (MC) accounts for about 1–7% of all BCs and generally affects elderly women, with a median age at diagnosis of 71 years [48].

Histologically, it is characterized by nests of cells in abundant extracellular mucinous material [49]. This special type usually expresses ERs and PRs [50].

On clinical examination, it appears as a palpable bump [51]. Axillary metastases are infrequent, being detected only in 13% of cases [50].

**Figure 4:**
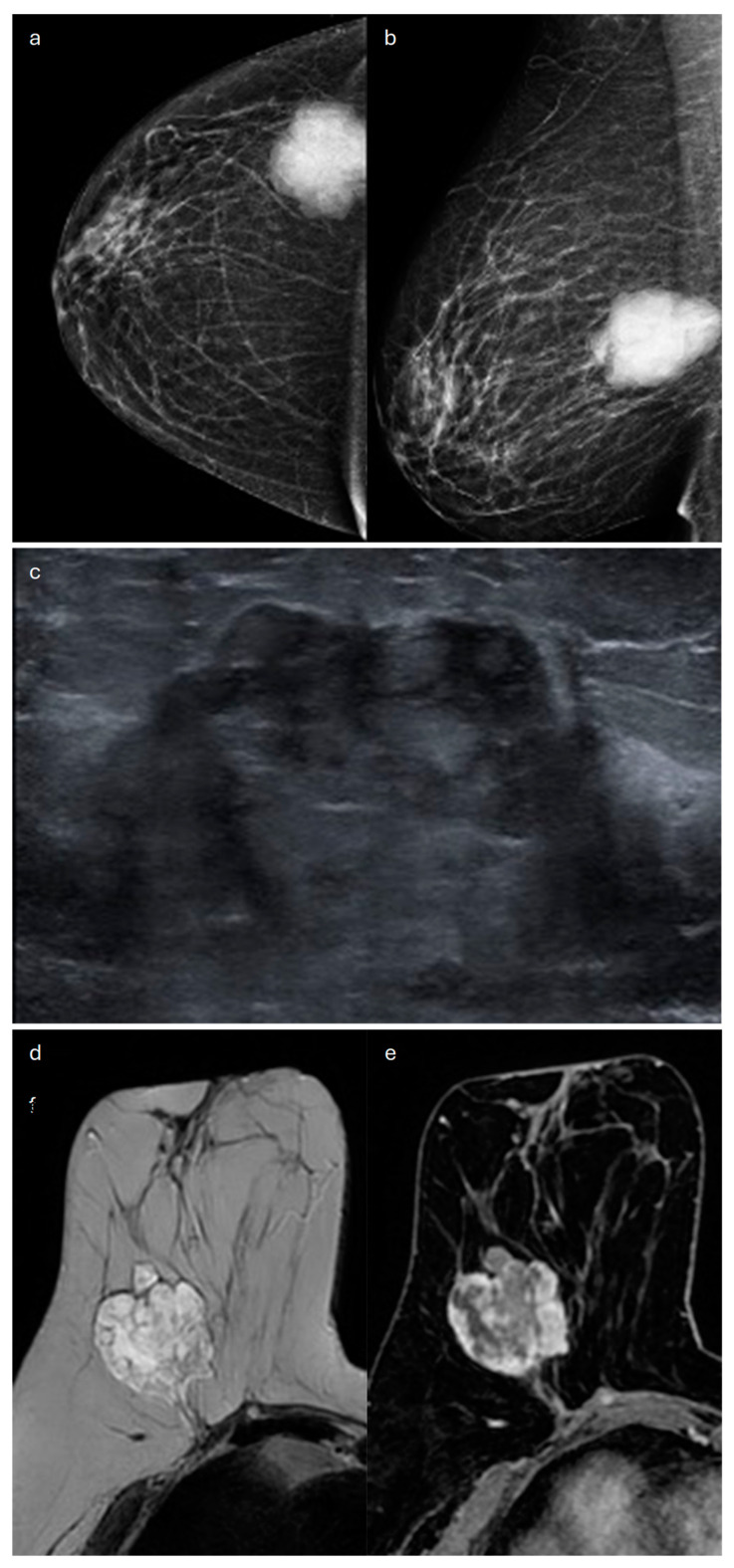
Mucinous carcinoma in a 58-year-old patient presenting with a right-breast lump on clinical examination. (**a**) Cranio-caudal and (**b**) medio-lateral oblique mammograms of the right breast show an oval-shaped mass with circumscribed margins at the junction of the upper and lower outer quadrants (**c**) corresponding to a hypoechoic lesion on ultrasound. (**d**) Axial T2-weighted MRI image shows multiple areas of high signal intensity within the mass corresponding to the mucinous content. (**e**) Axial T1-weighted fat-suppressed dynamic contrast-enhanced MRI image demonstrates a heterogeneous internal enhancement.

On MX, in more than 92% of cases, MC manifests as a solid mass that is round or oval-shaped, with circumscribed margins [52,53] (Figure 4).

A difference in the density of the lesion has been suggested in relation to the amount of mucin contained in it, emphasizing that the pure mucinous type (which contains mucin in more than 90% of the tumor mass) is more radiotransparent than the mixed type (in which mucin is present in less than 90% of the tumor mass) [54]. For this reason, it is not uncommon for pure MC to be undetectable on MX (about 20% of cases) [52]. With regard to margins, some authors suggest that pure MC can also present with non-circumscribed margins, i.e., it may be indistinct (48%) or microlobulated (28%), although this feature is usually suggestive of a mixed type [52,54]. Less frequently, pure MC may appear as a focal asymmetry (in about 11% of cases) [55]. Microcalcifications are not a typical finding of MC; if present, these are more indicative of a mixed type. Sometimes, MC may show round or amorphous calcifications [54,55].

On DBT, MC usually presents as a solid mass with circumscribed margins. Its detection, compared to that of MX, is improved in dense breasts due to the reduction of summation artefacts [56].

**Figure 5:**
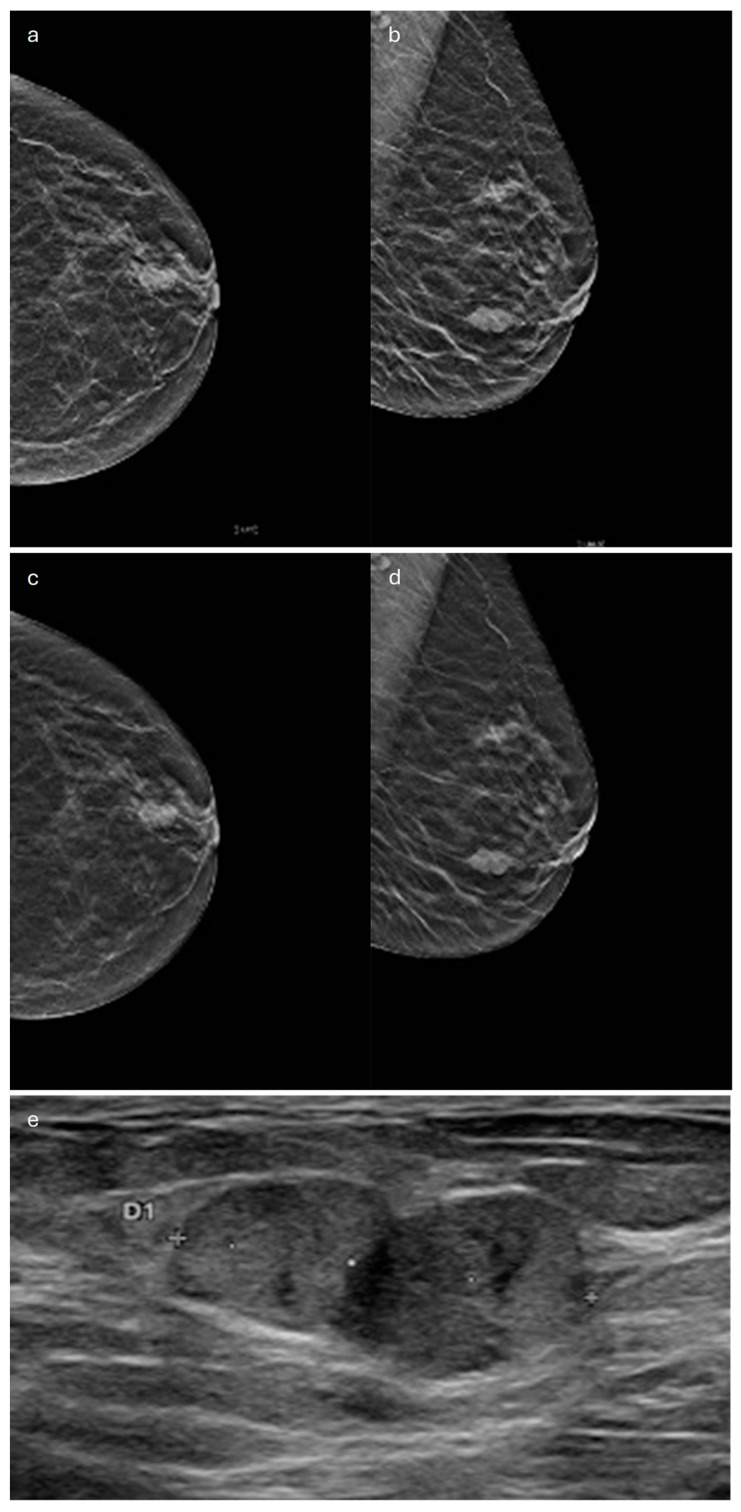
Mucinous carcinoma in a 56-year-old patient. (**a**) Cranio-caudal and (**b**) medio-lateral oblique 2D synthetic mammograms; (**c**) cranio-caudal and (**d**) medio-lateral oblique DBT images of the left breast show a solid mass with circumscribed margins (**e**) corresponding on US to a mass with circumscribed margins and acoustic enhancement that could mimic a benign lesion (e.g., fibroadenoma).

On US, MC is usually a well-circumscribed oval-shaped or round mass, parallel to the skin, that is often misdiagnosed as a benign lesion [52,54] (Figure 4 and Figure 5). In some cases, it may have an irregular shape and irregular/indistinct margins, with these features more commonly found in the mixed than in the pure type [52,54].

Pure MC is typically isoechoic, while mixed MC tends to be hypoechoic [57]. In 37.5% of cases, pure MC presents as a complex mass lesion with cystic and solid components [52]. Acoustic enhancement is commonly present [54] (Figure 5). There is not a typical pattern of vascularity: it may be absent or present within or adjacent to the mass [52,54]. Finally, in contrast-enhanced US, MC usually shows heterogeneous enhancement; in rare cases, no enhancement may be seen [51,58].

**Figure 6:**
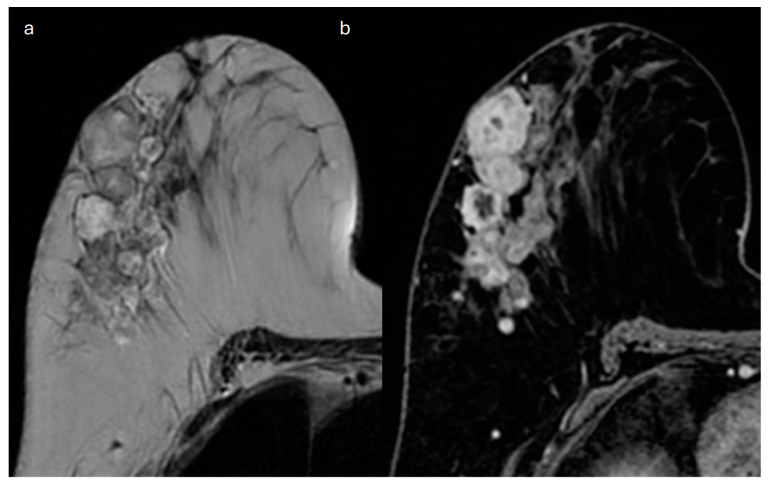
Breast MRI of a 45-year-old patient with a mucinous carcinoma of the right breast. (**a**) Axial T2-weighted MRI image shows multiple mass-like lesions tightly bound one to each other, one in continuity with the skin, characterized by internal areas of high signal intensity. (**b**) Axial T1-weighted fat-suppressed contrast-enhanced dynamic image demonstrates a heterogeneous internal enhancement.

MRI is useful for detecting tumor multifocality and multicentricity, which are seen in up to 10% of cases and are often undiagnosed through first-level imaging techniques [59]. Intense high signal on T2-weighted images, persistent on fat-saturated sequences, is a key feature of MC, which is typically homogeneous in the pure type and sometimes heterogeneous in the mixed type [60]. MC usually shows a low signal on DWI and a high signal on ADC map: this lack of diffusion restriction is caused by the high content of mucin compared to cellularity [60]. Mass enhancement is seen in more than 80% of cases, whereas NME is rare [54]. Even the contrast-enhancement pattern is influenced by the cellularity of the tumor: the pure mucinous hypocellular type typically shows an early rim enhancement that tends to get progressively diffuse and to be heterogeneous due to the distribution of the contrast medium through a high content of mucin [60]. In the pure mucinous hypercellular type and the mixed mucinous type, an intense early and persistent heterogeneous enhancement of the lesion is observed [61] (Figure 4, Figure 5 and Figure 6). In some rare cases, the pure mucinous type could show only delayed heterogeneous enhancement [61]. The pure mucinous type usually exhibits a type-I or -II contrast kinetics curve, whereas the mixed type typically shows a type-III curve [54].

## 5. Mucinous Cystadenocarcinoma

Mucinous cystadenocarcinoma (MCA) has been recognized as a rare special type of BC in the last WHO classification [5]. It is histologically characterized by cystic structures with papillae and a huge content of extracellular mucin [62]. It usually exhibits a triple-negative phenotype [62].

To our knowledge, less than 35 cases of MCA of the breast have been reported in the literature [63]. These data suggest a possible misdiagnosis of this kind of cancer, due to its peculiar features.

On clinical examination, it appears as a palpable mass, usually affecting post-menopausal women. Large lesions (over 4 cm) are frequent, being detected in 50% of cases [62], but nodal involvement is uncommon [64]. MCA usually has a good prognosis [63].

On MX or DBT, MCA mostly manifests as a multilobular or round/ovoidal dense mass, with well-defined margins [63,65,66,67]. Inner macrocalcifications or round to punctate microcalcifications have been reported [65,68].

**Figure 7:**
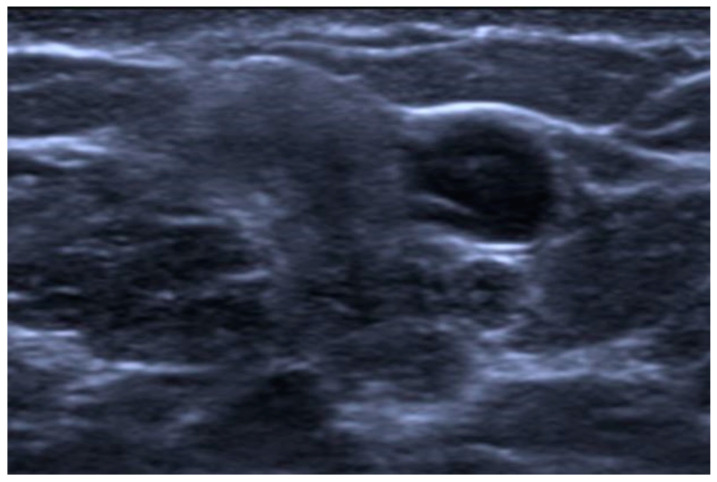
Mucinous cystadenocarcinoma in a 40-year-old patient. US image showing a complex cystic mass with a solid isoechoic component.

On US examination, MCA appears as an isoechoic or hypoechoic lesion with well-defined margins; an irregular-shaped mass or a complex cystic mass with solid components has been reported, too [62,65] (Figure 7).

Regarding MRI, only Seong et al. reported two cases of MCA and described them as complex irregular cystic and solid masses with intermediate signal on T2-weighted images, rim enhancement of the cystic component, and nodular heterogeneous enhancement of the solid mass, with persistent enhancement kinetics [65].

## 6. Medullary

The medullary subtype accounts for <1% of invasive BCs [69]. It mainly affects women aged from 45 to 55, but 10% of medullary tumors may be seen in women younger than 35 years [70,71]. BRCA1 mutation is associated with a higher risk of developing this tumor [71].

Histologically, it is characterized by a syncytial growth pattern, lymphoplasmacytic stromal infiltrate, nuclear pleomorphism, and histologic circumscription [51,72].

It clinically presents as a palpable breast lump, with axillary nodal metastases in 44% of cases [51]. This subtype typically has a triple-negative receptor pattern [71]. Nevertheless, the prognosis is favorable, with a 5-year overall survival of 89% [73].

**Figure 8:**
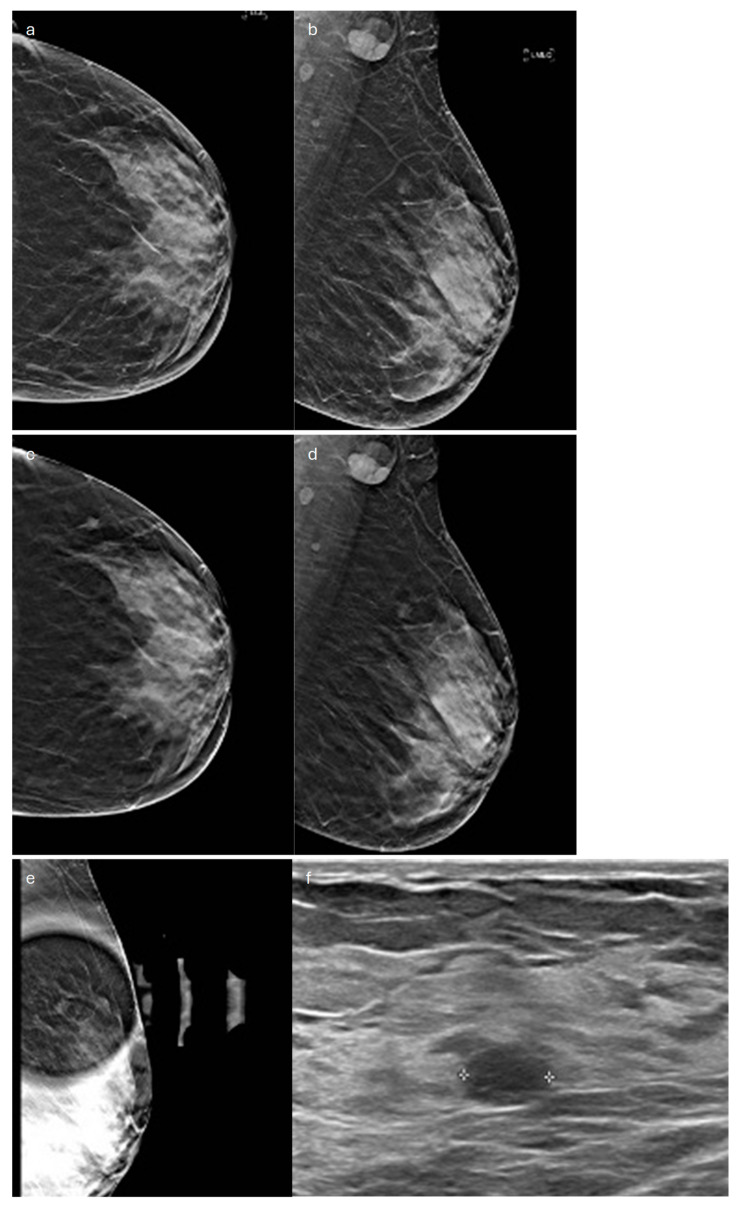
Medullary carcinoma in a 60-year-old patient. (**a**) Cranio-caudal and (**b**) medio-lateral oblique 2D synthetic mammograms, (**c**) cranio-caudal DBT image, (**d**) medio-lateral oblique DBT image and (**e**) spot compression view of the left breast showing an oval-shaped opacity in the upper-outer quadrant (**f**) corresponding to an oval-shaped homogeneous hypoechoic mass on ultrasound.

On MX or DBT, it usually shows benign imaging features, presenting as a round or oval-shaped, circumscribed, dense mass, similarly to MC [52] (Figure 8). Rarely, it may exhibit indistinct or irregular margins [73]. The presence of the halo sign has been reported [74]. Small, round, or amorphous microcalcifications may be associated with the mass [74].

The US appearance of this subtype consists of a hypoechoic homogeneous round or oval-shaped circumscribed mass parallel to the skin [53,72] (Figure 8). Less commonly, the lesion may be heterogeneous and irregularly shaped, with indistinct margins; shadowing may be seen in some cases [75]. On color Doppler, rim vessels are frequently present [75]. On contrast-enhanced ultrasound (CEUS), medullary tumors show homogeneous enhancement and high average peak-intensity values [76].

MRI is a crucial tool with which to detect multifocality or multicentricity (10% of cases) [51]. The majority of medullary carcinomas display mass enhancement that is round, oval, or lobular in shape, typically with circumscribed margins [51,72]. They are characterized by iso- or slight hyper-intensity on T2-weighted images, occasionally associated with a hypointense rim representing the presence of a capsule. When edema or hemorrhage is present within the lesion, hyperintensity may be seen [77]. Restricted diffusion in the DWI/ADC map is usually present [78]. The early enhancement pattern tends to be homogeneous, but it may become heterogeneous in case of inner necrosis and cystic degeneration [51]. Late rim enhancement is typical, probably due to lymphoplasmacytic reaction [79]. The contrast kinetics include type-II/IIII curves [77,79].

## 7. Cribriform

Invasive cribriform carcinoma (ICC) is a rare type of invasive BC (reported incidence 0.3–3.5%) [80]. It is classified into pure and mixed types, with >90% of the mass in the pure type showing invasive cribriform morphology [80]. It is more common in postmenopausal women, with a median age of 46.5 years for the pure type and 54 years for the mixed type.

ICC is histologically characterized by a cribriform growth pattern of the cells, which usually express ERs and PRs, while amplification of HER-2 is rare [81]. The pure type has a better prognosis than the mixed one, with 10-year survival of about 90%. Axillary nodal involvement is very rare [80,81].

The MX appearance of both pure and mixed ICC is mainly characterized by an irregular-shaped opacity with spiculated margins and high density, or, less commonly, by an oval-shaped opacity with circumscribed margins or associated microcalcifications (mostly pleomorphic) [80,82,83].

On US, ICC usually shows as an irregular-shaped, hypoechoic lesion with spiculated margins. Some features, such as orientation parallel to the skin and lack of posterior acoustic shadowing, are generally described as specific to pure ICC [80]. By contrast, Balci et al. reported a nonparallel orientation as the most frequent in both types [83].

In the few cases reported in the literature on MRI, ICC mostly presents as a mass lesion with an irregular shape and non-circumscribed margins (irregular or spiculated) and heterogeneous enhancement; rarely, a segmental NME or a round mass with rim enhancement has been described [82,83].

## 8. Papillary and Micropapillary

Papillary carcinoma (PC) and invasive micropapillary carcinoma (IMPC) are two distinct entities, with different behaviors and prognoses.

PC accounts for 1–2% of BCs. It usually affects post-menopausal women and presents as a palpable mass [84]. In 22–34% of cases, bloody nipple discharge is present [85]. Invasive PC may be intracystic, in which case it grows into a dilated duct, or solid; in both cases, the prognosis is better than that of IDCa and metastases are rare [84,85].

Invasive PC is histologically characterized by the presence of papillae with a fibrovascular core in dilated ducts [62].

**Figure 9:**
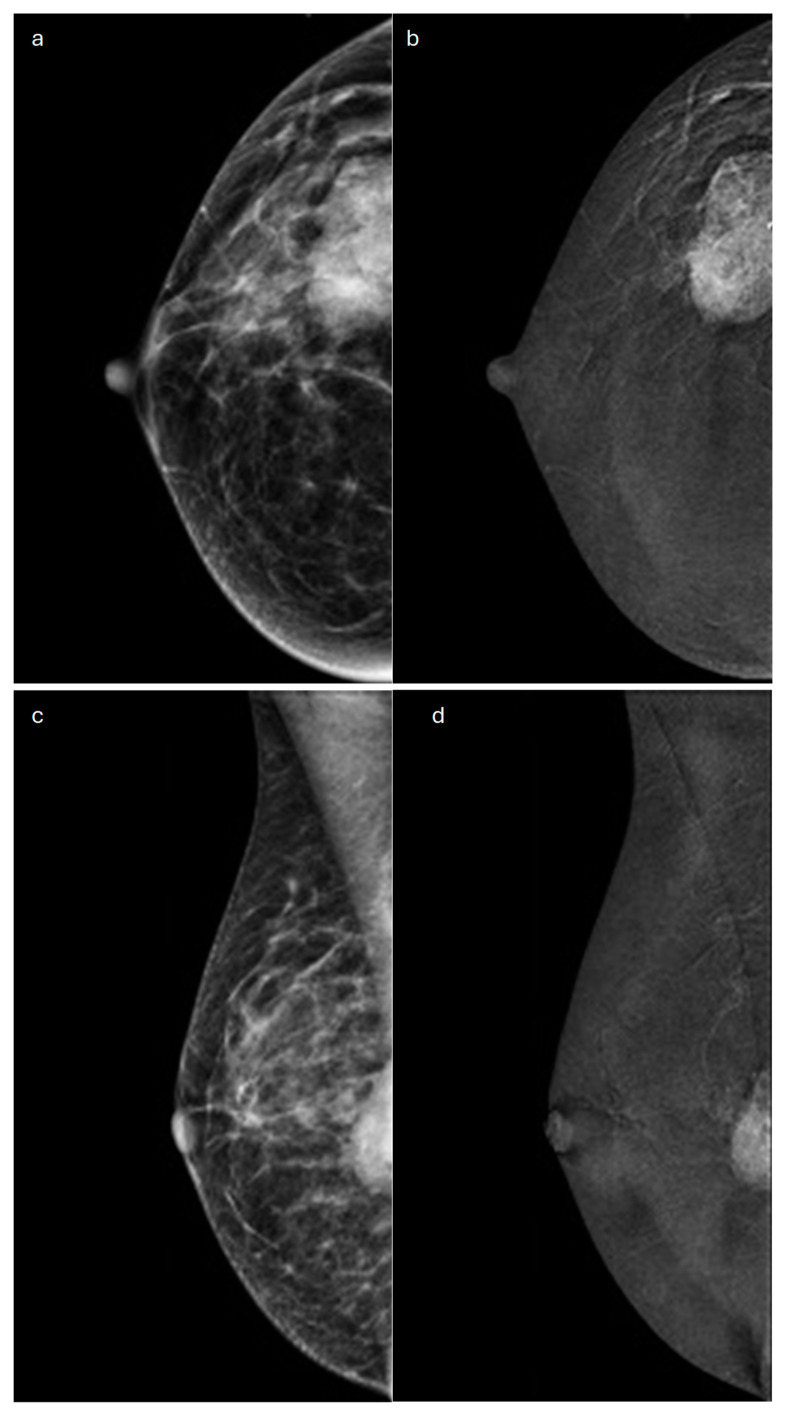
Papillary carcinoma in a 49-year-old patient. Contrast-enhancement mammography: low-energy (**a**) cranio-caudal and (**c**) medio-lateral oblique images of the right breast show a high-density mass, apparently oval-shaped (only partially included), at the junction of the upper and lower outer quadrants. Some microcalcifications are visible within the mass. (**b**–**d**) The mass corresponds to an area of enhancement on the recombined images.

**Figure 10:**
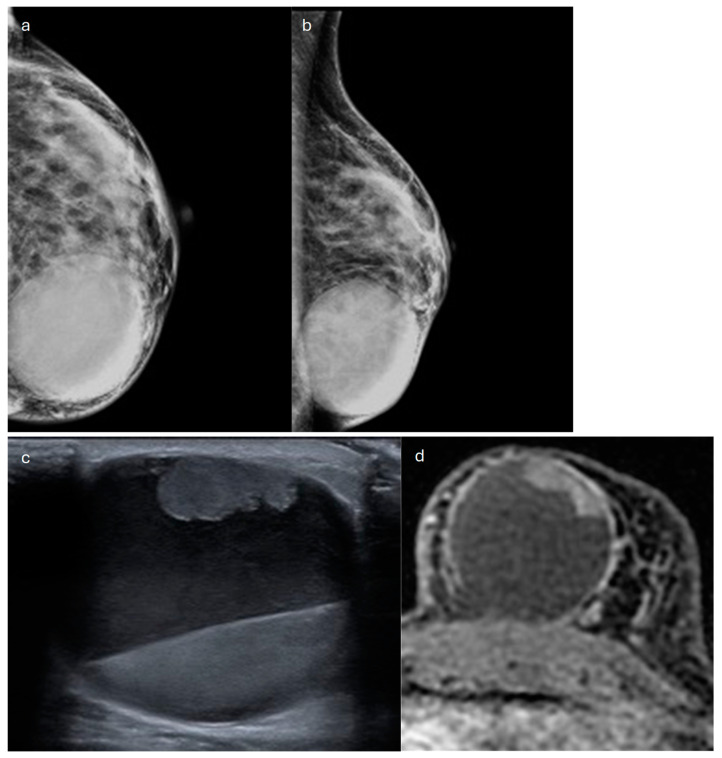
Papillary carcinoma in a 59-year-old patient. (**a**) Cranio-caudal and (**b**) medio-lateral oblique mammograms of the left breast show a round opacity in the lower-inner quadrant (**c**) corresponding on ultrasound to a complex cystic mass with a solid anterior component. A fluid−fluid level is visible inside the cyst due to the bleeding produced during the biopsy procedure. (**d**) Axial 3D gradient echo T1-weighted post-contrast MRI image of the left breast shows the enhancement of the solid component of the lesion.

On MX and DBT, PC usually presents as a round or oval-shaped opacity with lobulated margins (Figure 9 and Figure 10). Microcalcifications may be present but are infrequent [84,85].

On US, PC usually appears as hypoechoic solid or complex cystic and solid mass, which can be intraductal, single, or multiple; associated ductal dilatation is frequently observed [84,85] (Figure 10).

On MRI, PC may have variable appearances and kinetic curves, making it difficult to distinguish it from a benign lesion. It may present as an enhancing solid mass or a complex cyst and is usually heterogeneously hyperintense on T2-weighted images and hypointense on T1-weighted images [84,85] (Figure 10). Although ADC values have been suggested as a tool to discriminate between benign papillary lesions (high ADC values) and malignant ones (low ADC values) [86], care needs to be taken with DWI because benign papillary lesions represent a typical example of false positives for DWI [87].

IMPC accounts for <5% of BCs [88] and has a mean age at diagnosis of 59.5 years [89]. Histologically, IMPC usually displays an “inside-out” growth pattern [62], and tumor cells mostly express ERs and PRs [90].

IMPC commonly manifests as a palpable mass [91]. Axillary lymphadenopathy has been reported as a common finding, observed in a variable percentage of cases (38–67%) [90,91,92].

Nangong et al. and Jones et al. described IMPC’s main MX features: high-density opacity with irregular shape and spiculated margins [90,92]. In contrast, Günhan-Bilgen et al. found a round or oval-shaped mass in most of their cases [93]. The presence of calcifications was also reported, with these mainly described as punctate with scattered distribution or as pleomorphic [90,91,94].

**Figure 11:**
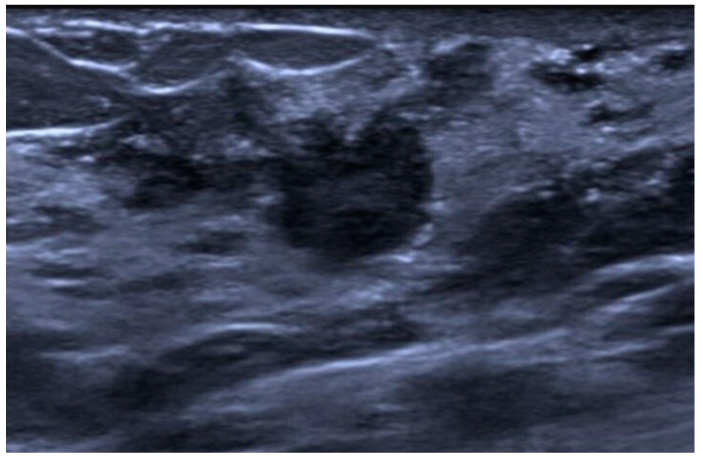
Micropapillary carcinoma in a 75-year-old patient. US image shows a round hypoechoic mass with non-circumscribed margins.

Its main US features are hypoechogenicity, irregular shape, and spiculated margins [90,92,95]; some authors have also described an isoechoic pattern in 50% of masses [96] (Figure 11). On color Doppler, IMPC frequently shows inner blood flow [90,95].

The most common MRI features of IMPC are irregular shape, spiculated margins, heterogeneous internal enhancement, and kinetic curves with type-II/III patterns [90,92,94].

## 9. Apocrine

The term “apocrine pattern” refers to a pathological finding characterized by the presence of enlarged cells with a cuboidal or columnar shape, eosinophilic cytoplasm, apical snouts or blebs, and round nuclei. Apocrine changes have been recognized in benign, atypical, and malignant lesions of the breast [97]. Although the benign spectrum is routinely found on histopathological examinations, atypical and malignant apocrine lesions are rare.

The 2019 WHO classification of breast tumors stated that an essential criterion to define apocrine carcinoma (AC) is the presence of >90% of the distinctive morphology in cancer cells. Desirable criteria include cells that are ER-negative and androgen-receptor-positive [98]; if these criteria are strictly used to define AC, it represents less than 1% of all BCs [99]. DCIS variants include apocrine ductal carcinoma in situ (ADCIS), in which tumoral cells have an apocrine morphology; nevertheless, there are no defined criteria to evaluate its extent or grading [100]. It is not always easy to make a distinction between ADCIS and atypical apocrine lesions because of their possible association with nuclear atypia and high cellularity. High-grade ADCIS can present as necrosis, calcification, or periductal changes. ADCIS is usually associated with intermediate- or high-grade invasive carcinomas [101].

The mean age of the patients at diagnosis is 58.5 years [101].

To our knowledge, only a few studies have investigated the imaging features of AC, with these studies mainly consisting of case series or case reports. All reported results demonstrate that the imaging features of AC or ADCIS are not different from those of NST-ICD or DCIS.

**Figure 12:**
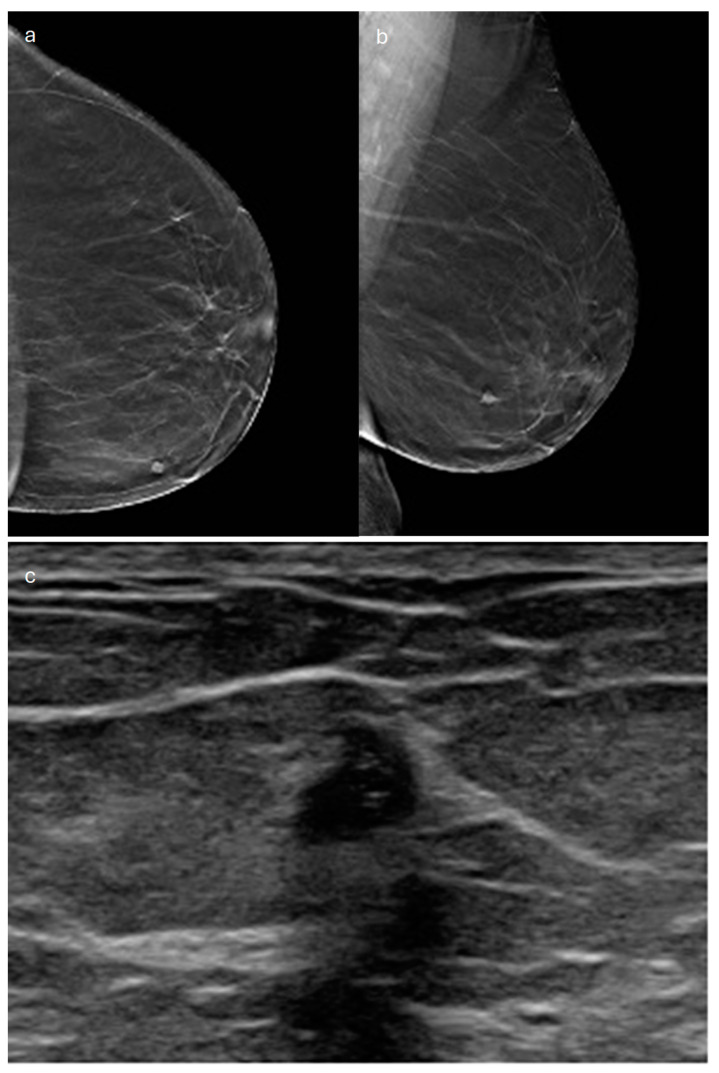
Apocrine carcinoma in a 61-year-old patient. (**a**) Cranio-caudal and (**b**) medio-lateral oblique DBT images of the left breast show a small, irregularly shaped opacity in the lower-inner quadrant (**c**) corresponding to a hypoechoic mass with non-circumscribed margins on ultrasound.

On MX, AC predominantly appears as an irregular or oval-shaped high-density mass with ill-defined margins (i.e., indistinct or spiculated) [102,103,104,105] (Figure 12); rarely, as a focal asymmetry [101,104]. AC is often associated with microcalcifications, which are usually described inside the opacity [101,102]; sometimes, it can present as microcalcifications only [101].

Similarly, on US, AC shows as an irregular or oval-shaped hypoechoic mass with either non-circumscribed or well-circumscribed margins, mainly with a parallel orientation [101,104] (Figure 12). An uncommon presentation can be as a cyst or a complex cyst with inner solid components or thick septa [101,105,106].

In the few reports in the literature, the MRI appearance of AC is characterized by a mass lesion with early rim or heterogeneous enhancement and a wash-out pattern in the delayed phase [101,103,104].

## 10. Metaplastic

Metaplastic BC (MBC) has a unique histology that differentiates it from other subtypes: it is constituted of two or more malignant cellular types, i.e., epithelial and mesenchymal cells [107].

Metaplastic changes can be different in type and degree, resulting in various histopathologic subtypes [108]. The morphological features on pathologic examination helps to differentiate between the metaplastic types with different epithelial populations. On the other hand, an immunohistochemistry analysis is needed when the subtype is characterized by a mesenchymal component, highlighting the expression of both epithelial (cytokeratin) and mesenchymal (vimentin) cell markers [109].

The mean age at diagnosis in the literature ranges from 51.6 to 58.5 years [107,110,111]. Toumi et al. reported a 5-year disease-free survival between 40% and 84% and a 5-year overall survival between 64 and 83% [110].

MBC usually manifests as a clinically palpable mass due to its large dimensions at diagnosis. Pezzi et al. examined the biological behavior of 892 cases of MBC, finding that the majority of them (~70%) were >20 mm and that, in about 20% of cases, they were even larger than 5 cm; these percentages are definitely higher than those associated with patients with ICD [112]. Also, Donato et al. and Jia et al. reported median sizes at diagnosis of 27 mm (range 11–100 mm) and 34 mm, respectively [107,113].

**Figure 13:**
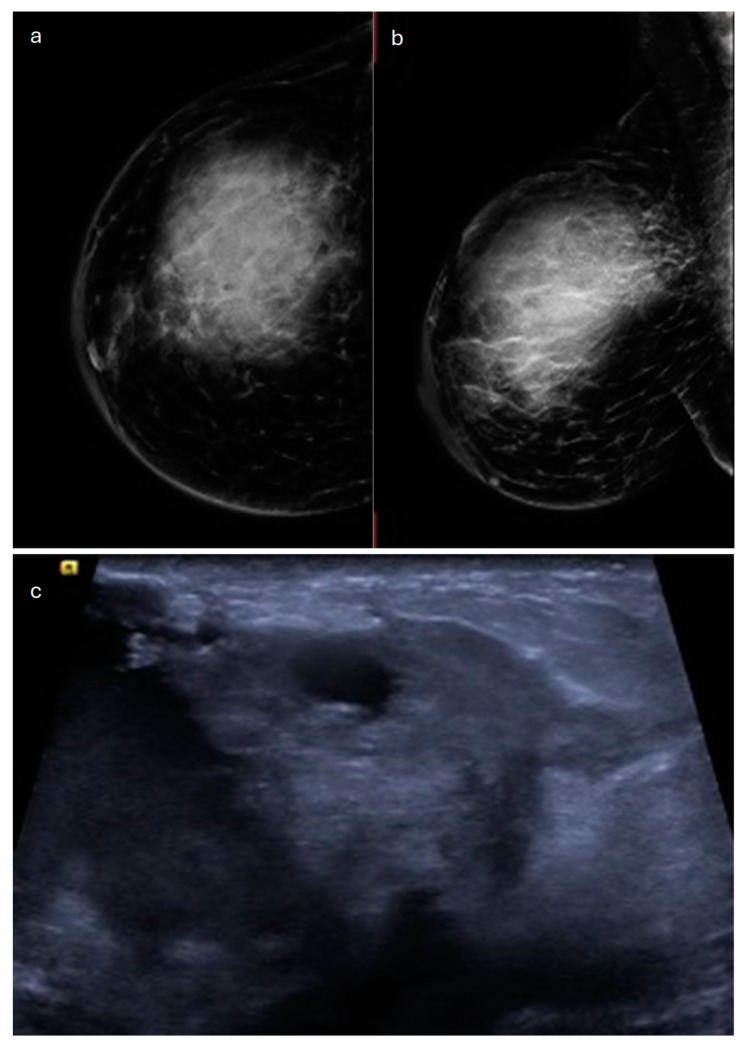
Metaplastic carcinoma in a 43-year-old patient. (**a**) Cranio-caudal and (**b**) medio-lateral oblique mammograms of the right breast show a high-density mass with indistinct margins predominantly located in the upper-outer quadrant and (**c**) corresponding to an iso-hypoechoic mass with partially indistinct margins and cystic components on ultrasound.

**Figure 14:**
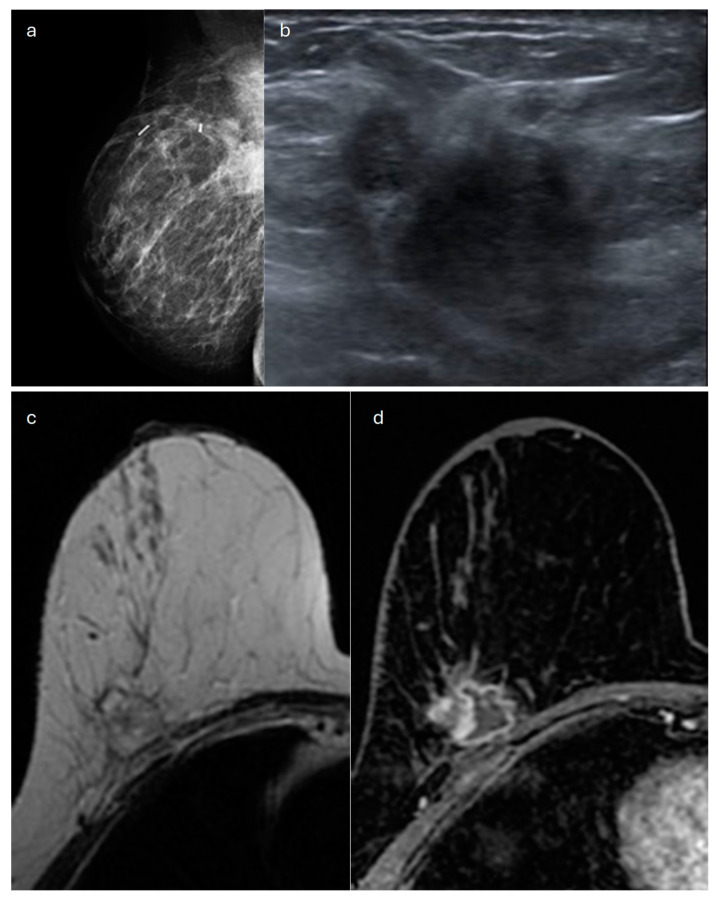
Metaplastic carcinoma in a 40-year-old patient. (**a**) Medio-lateral oblique mammogram of the right breast shows a high-density mass, only partially included, with indistinct margins in the upper quadrant in the posterior third. Surgical clips from a previous surgery are visible. The cranio-caudal projection is not presented because the mass was not visible on it due to its position. (**b**) On ultrasound, it corresponds to a hypoechoic mass with irregular margins. (**c**) Axial T2-weighted MRI image shows high-signal-intensity areas within the mass corresponding to necrotic components, and (**d**) axial 3D gradient echo T1-weighted post-contrast MRI image demonstrates a ringlike uptake.

MBC usually presents on MX as an oval-shaped opacity with high density and circumscribed or indistinct margins [107,114] (Figure 13 and Figure 14).

Concerning the US features, MBC typically manifests as an oval-shaped mass with circumscribed margins and posterior acoustic enhancement [113,115,116], while indistinct margins are less frequently described [114,116] (Figure 13 and Figure 14).

The most common MRI appearance of MBC is that of a mass lesion with a typical central hyperintensity on T2-weighted images, which is probably linked to the necrotic component [107,111,117], with diffusion restriction on DWI/ADC maps [111]. After contrast-medium administration, MBC usually presents with heterogeneous enhancement or rim enhancement and a type-2 or -3 kinetic curve [117] (Figure 14).

## 11. Neuroendocrine Carcinoma

Breast carcinoma with neuroendocrine differentiation is a rare type of breast neoplasia, accounting for 2–5% of all BCs [118]. Post-menopausal women are the most frequently affected [119].

In 2019, WHO revised its classification using the term “neuroendocrine neoplasms” (NEN) to refer to all tumors with dominant neuroendocrine differentiation. Invasive carcinomas with less than 10% neuroendocrine morphology are considered IDC-NST. When 10% or more of the tumor has a neuroendocrine morphology, the lesion can be classified as NEN. The diagnosis of NENs is typically made with the use of immunohistochemistry, which highlights the presence of markers such as synaptophysin, chromogranin A, and neuron-specific enolase [119]. NENs are divided into two main categories: well-differentiated neuroendocrine tumors (NETs) and poorly differentiated neuroendocrine carcinomas (NECs). Neuroendocrine tumors are further divided into grade 1 (carcinoid-like) and grade 2 (atypical carcinoid-like); on the other side, NECs include small-cell and large-cell types [120,121]. NECs can be considered a subtype of luminal carcinomas that demonstrate lower rates of PIK3CA mutations; this particularity might contribute to their unfavorable prognosis [119]. Although the prognosis of NECs remains controversial, recent studies demonstrated that neuroendocrine differentiation is an independent negative prognostic factor when compared to NST-IDC [119]. Most of the cases of NECs reported in literature are ERs- and PRs-positive and HER-2-negative [119,122,123].

Given their rarity, there are not many studies on this subject, and its radiological features are mainly described in case reports.

Patients usually report the presence of a palpable mass and nipple discharge at clinical examination [122,124].

On MX, NECs usually present as oval-shaped or round masses with margins that can be either circumscribed or non-circumscribed, so they can mimic both triple-negative BC and benign lesions [122,124,125]. Calcifications are uncommon [112]. In some cases, NECs can appear as large masses with associated skin thickening and axillary lymphadenopathies [123,126]. On US, NECs are usually described as hypoechoic masses with irregular morphology and indistinct margins [122,123,124].

In MRI, NECs typically present with features highly indicative of malignancy: mass lesions with irregular shape, non-circumscribed margins, and washout kinetics are usually described [122,124].

## 12. Rare and Salivary-Gland-Type Tumors

According to the WHO, there is a subset of BC that display the same characteristics as salivary-gland tumors in terms of morphology, immunophenotype, and molecular genetics. Despite being usually triple-negative, these carcinomas have a better prognosis than NST ones [127]. This category includes secretory carcinoma, adenoid cystic carcinoma, acinar cell carcinoma, mucoepidermoid carcinoma, polymorphous adenocarcinoma, and tall cell carcinoma with reversed polarity [5,127]. In this review, we will focus on secretory and adenoid cystic carcinomas.

Secretory carcinoma (SC) is a rare BC subtype, accounting for less than 1% of BCs. It typically affects women in their 50’s (with a median age of 53–56 years at diagnosis), but it has been reported also in patients younger than 10 years old [128,129].

Histopathologically, SC is characterized by cells with low-grade nuclei, a small amount of cytoplasm, and intra- and extra-cellular secretory material arranged in cystic, glandular, and solid nests separated by collagen bands; this creates a characteristic “honeycomb” pattern [129,130].

Initially, SC was thought to be negative for ERs, PR, and HER2 [131], while a more recent study by Jacob et al. on 246 SCs from the National Cancer Database in the United States found that 64% of SCs were classified as ER-positive [128]. However, it should be noted that their positivity for ERs and PRs is weak, with these receptors being expressed in a reduced number of cells, as shown by Hoda et al. [129].

Despite this unfavorable receptor profile, SC has an excellent overall prognosis: the cancer-specific survival is over 90% at 10 years [128,129].

Given the rarity of this subtype, there are few works on this topic, and these are almost entirely based on case reports. Clinically, SCs usually present as a palpable bump [128,129]. In about 35% of cases, there are positive lymph nodes at diagnosis, whilst metastatic spread is rare, being present in only 2% of cases [128,131].

The MX and DBT features do not allow characterization of the lesion, which can also be mistaken for a benign formation [132,133].

US examination typically shows a benign-looking hypoechoic or isoechoic mass with an oval, round, or tubular shape [133,134]. SC can present as a single lesion or as multiple nodules [134].

On MRI, it appears as a complex cystic mass on T2-weighted images, with an early wash-out enhancement [134].

Adenoid cystic carcinoma (AdCC) is a rare BC subtype, accounting for less than 0.1% of BCs. It is associated with a better prognosis than extra-breast AdCCs, with axillary nodal involvement and distant metastases being rare [135].

Histopathologically, AdCC is characterized by the presence of both an epithelial and a basaloid/myoepithelial component. It has many histologic variants, which are here listed in order of aggressive clinical behavior: the classic form, the solid variant with basaloid features, and the AdCC with high-grade transformation, the last being associated with a poorer prognosis because of the coexistence of an AdCC with a high-grade triple-negative BC [127].

On MX and US, AdCC typically appears as a round or oval-shaped mass with well-circumscribed margins, often resembling benign features [133]. Moreover, on US, AdCC usually shows a parallel orientation and, in about 63% of cases, iso- or hyperechogenicity; in a small number of cases, a cystic-like appearance may be observed as well [135].

**Figure 15:**
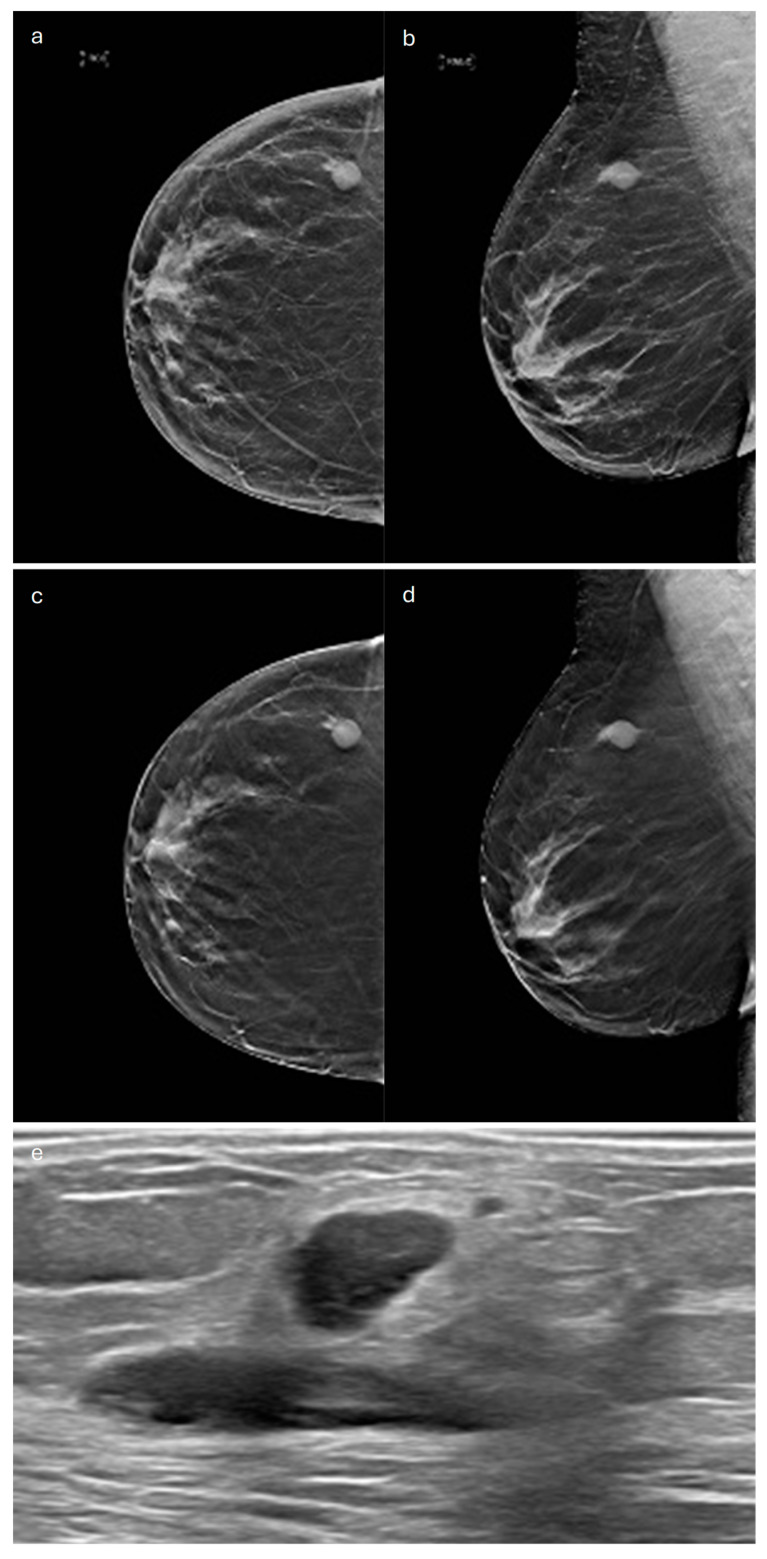

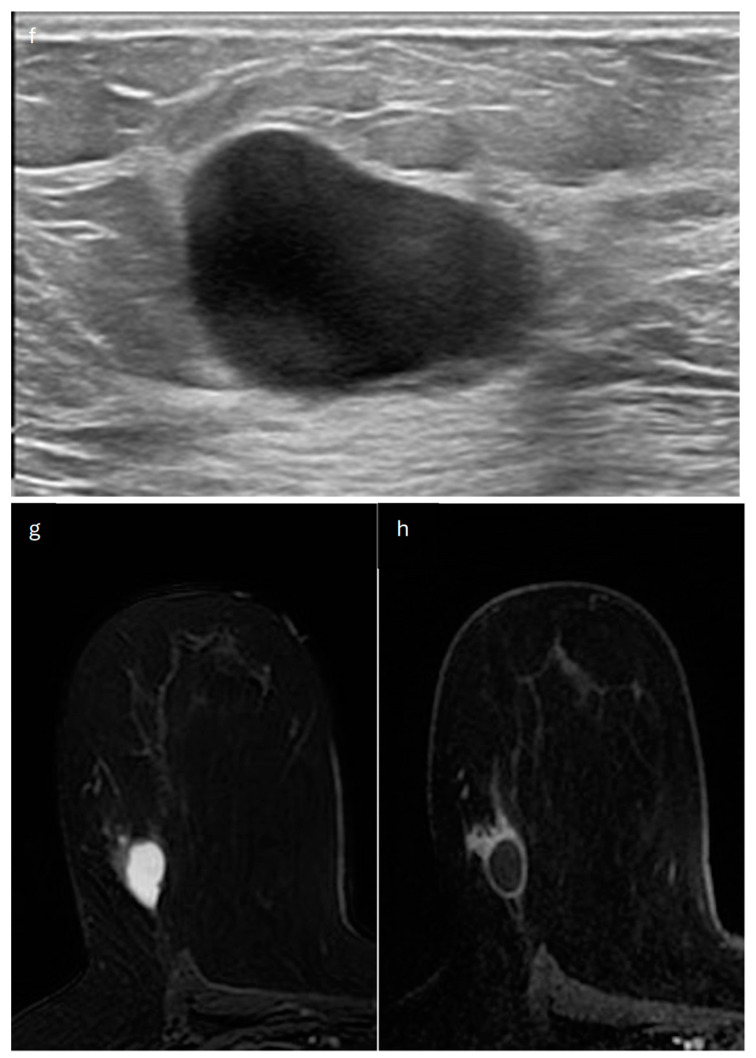
Solid-basaloid variant of adenoid cystic carcinoma. (**a**) Cranio-caudal and (**b**) medio-lateral oblique 2D synthetic mammograms, (**c**) cranio-caudal and (**d**) medio-lateral oblique DBT images of the right breast show a round opacity with circumscribed margins in the upper-outer quadrant (**e**,**f**) corresponding to a hypoechoic mass with cystic components on ultrasound. (**g**) Axial T2-weighted fat-suppressed MRI image shows high signal from the cystic components, and (**h**) axial T1-weighted fat-suppressed dynamic contrast-enhanced MRI image demonstrates the enhancement of the solid component.

On MRI, a mass lesion with high signal in T2w, restricted diffusion in DWI/ADC, and homogeneous enhancement is a typical finding [135]. Large masses may show a late-enhancing internal septum [136] (Figure 15).

## 13. Conclusions

BC encompasses a wide variety of rare histological subtypes that differ from the most common IDC. These special subtypes have distinctive clinical presentations, radiological appearances, and prognoses. Radiologists play a central role in the diagnosis of BC, so they should be aware of all these entities and their specific multimodal-imaging features (Table 1) in order to correctly recognize them and properly direct patients to the correct diagnosis and treatment, thus avoiding delays and mistakes in the diagnostic−therapeutic process and anxiety in patients. However, radiological characteristics represent an important clue that must always be related to clinical findings and histopathology if they are to be used to correctly understand the disease and guide subsequent management decisions.

## Figures and Tables

**Table 1:** Main multimodal-imaging findings of each special type of breast cancer.

Type	Mammography	Ultrasound	MRI
Lobular	- Mass with spiculated/irregular or ill-defined margins - Asymmetric density with radiopacity similar to that of normal breast parenchyma - Architectural distortion (better detected with DBT) - Undetectable in 19–43% of cases On CEM: - Oval-shaped or irregularly shaped mass lesion with irregular margins and heterogeneous internal enhancement - In a minority of cases, NME	- Hypoechoic mass with an irregular shape, indistinct margins, and posterior acoustic shadowing - Diffuse infiltrative inhomogeneous hypoechoic pattern with indistinct margins and no shadowing - Undetectable in 10% of cases Rarely: - Isoechogenicity of the mass - Smooth, lobulated, or well-circumscribed mass with parallel orientation and peripheral vascularity	- Irregular mass with spiculated or irregular margins with restricted diffusion and heterogeneous contrast enhancement, with no specific kinetic curves - NME (ductal, segmental, regional, or diffuse pattern) - 93–95% sensitivity in detecting multifocality and multicentricity Rarely: - Multiple enhancing foci around the main lesion
Tubular	- Irregular mass with spiculated margins - On DBT, mass with long spicules or architectural distortion	- Hypoechoic solid mass with non-circumscribed margins and posterior acoustic shadowing (similar to IDC-NST)	- Irregular-shaped mass with non-circumscribed margins and low-to-intermediate signal on T2-weighted images and heterogeneous enhancement, with a persistent kinetic curve Rarely: - Non-enhancing lesion - High-signal-intensity lesion with hypointense internal septation-like appearance on T2-weighted images
Mucinous	- Round or oval-shaped opacity with circumscribed margins Rarely: - Opacity with indistinct or microlobulated margins (mixed type) - Radiotransparency (pure type) - Focal asymmetry	- Well-circumscribed oval-shaped or round mass, parallel to the skin, with acoustic enhancement (often misdiagnosed with a benign lesion) - Isoechoic (pure type)/hypoechoic mass (mixed type) Rarely: - Irregularly shaped lesion with irregular/indistinct margins (mixed) - Complex mass lesion with cystic and solid components (pure)	- Intense high signal on T2-weighted images, homogeneous (pure)/sometimes heterogeneous (mixed) - Low signal on DWI and high signal on ADC map - Heterogeneous, intense early and persistent mass enhancement (mixed)/rim enhancement that becomes progressively diffuse (pure) - No specific kinetic curves
Mucinous cystadenocarcinoma	- Multilobular or round/ovoidal dense mass with well-defined margins - Inner macro/micro- calcifications may be present	- Isoechoic or hypoechoic lesion with well-defined margins - Irregularly shaped mass, complex cystic mass with solid components	- Complex irregular cystic and solid mass with intermediate signal on T2-weighted images - Rim enhancement of the cystic component and nodular heterogeneous enhancement of the solid component - Persistent enhancement kinetics
Medullary	- Round or oval-shaped, circumscribed, high-density opacity Rarely: - Opacity with indistinct or irregular margins - Associated small, round, or amorphous calcifications - Halo sign has been reported	- Hypoechoic, homogeneous round or oval-shaped circumscribed mass, parallel to the skin Rarely: - Heterogeneous, irregular-shaped lesion, with indistinct margins - Shadowing may be seen	- Mass enhancement - Round, oval-shaped, or lobular mass lesion with circumscribed margins - Iso- or slight hyper-intensity on T2-weighted images, occasionally with hypointense rim (capsule) - Restricted diffusion on DWI/ADC map - Late rim enhancement - Type-II/IIII kinetics curves
Cribriform	- Irregular-shaped opacity with spiculated margins and high density Rarely: - Oval-shaped opacity with circumscribed margins or associated microcalcifications	- Irregular-shaped, hypoechoic lesion with spiculated margins	- Mass lesion with irregular shape and non-circumscribed margins (irregular or spiculated), with heterogeneous enhancement Rarely: - NME with segmental distribution - Round mass, with rim enhancement
Papillary and Micropapillary	Papillary: - Round or oval-shaped opacity with lobulated margins, rarely associated with microcalcifications Micropapillary: - High-density opacity with irregular shape and spiculated margins - Round or oval-shaped mass - Calcifications may be present	Papillary: - Hypoechoic solid or complex cystic-solid mass (intraductal, single, or multiple), often associated with ductal dilatation Micropapillary: - Hypoechogenicity, irregular shape, and spiculated margins - Isoechoic pattern	Papillary: - Enhancing solid mass or complex cyst, usually heterogeneously hyperintense on T2-weighted images and hypointense on T1-weighted images Micropapillary: - Irregular-shaped mass with spiculated margins, heterogeneous internal enhancement with type-II/III kinetic curves
Apocrine	- Irregularly shaped or oval-shaped high-density mass with ill-defined margins (indistinct or spiculated) - Often associated with inner microcalcifications Rarely: - Focal asymmetry	- Irregularly shaped- or oval-shaped hypoechoic mass with either non-circumscribed or well-circumscribed margins, mainly with a parallel orientation Rarely: - Cyst or complex cyst with inner solid components or thick septa	- Mass lesion with early rim or heterogeneous enhancement followed by a wash-out pattern in the delayed phase
Metaplastic	- Oval-shaped opacity with high density and circumscribed or indistinct margins	- Oval-shaped mass with circumscribed margins and posterior acoustic enhancement - Indistinct margins are less frequently described	- Mass lesion with central hyperintensity on T2-weighted images, diffusion restriction on DWI/AD, and heterogeneous enhancement or rim enhancement and type II/III kinetic curves
Neuroendocrine	- Oval-shaped or round mass with circumscribed or non-circumscribed margins - Calcifications are uncommon	- Hypoechoic mass with irregular morphology and indistinct margins	- Mass lesion with irregular shape, non-circumscribed margins, and washout kinetics
Secretory	- MX and DBT features of benignity	- Benign-looking hypoechoic or isoechoic mass with an oval, round, or tubular shape	- Complex cystic mass on T2-weighted images with an early wash-out enhancement
Adenoid cystic	- Round or oval-shaped mass with well-circumscribed margins	- Round or oval-shaped well-defined hyperechoic or isoechoic mass with parallel orientation Rarely: - Cystic-like appearance	- Mass lesion - High signal on T2-weighted images - Restricted diffusion on DWI/ADC map - Homogeneous enhancement

## Data Availability

No new data were created.

## References

[1] Rakha E.A., Reis-Filho J.S., Baehner F., Dabbs D.J., Decker T., Eusebi V., Fox S.B., Ichihara S., Jacquemier J., Lakhani S.R. (2010). Breast Cancer Prognostic Classification in the Molecular Era: The Role of Histological Grade. Breast Cancer Res. BCR.

[2] Ellis I.O., Galea M., Broughton N., Locker A., Blamey R.W., Elston C.W. (1992). Pathological Prognostic Factors in Breast Cancer. II. Histological Type. Relationship with Survival in a Large Study with Long-Term Follow-Up. Histopathology.

[3] Jenkins S., Kachur M.E., Rechache K., Wells J.M., Lipkowitz S. (2021). Rare Breast Cancer Subtypes. Curr. Oncol. Rep..

[4] Hwang H., Sahoo S., Saluja K. (2022). Invasive Breast Carcinoma of No Special Type, Microinvasive Carcinoma, Tubular Carcinoma, and Cribriform Carcinoma. A Comprehensive Guide to Core Needle Biopsies of the Breast.

[5] World Health Organization (2019). WHO Classification of Tumours: Breast Tumours.

[6] Kufel J., Bargieł K., Koźlik M., Czogalik Ł., Dudek P., Jaworski A., Cebula M., Gruszczyńska K. (2022). Application of Artificial Intelligence in Diagnosing COVID-19 Disease Symptoms on Chest X-rays: A Systematic Review. Int. J. Med. Sci..

[7] Ahn J.S., Shin S., Yang S.-A., Park E.K., Kim K.H., Cho S.I., Ock C.-Y., Kim S. (2023). Artificial Intelligence in Breast Cancer Diagnosis and Personalized Medicine. J. Breast Cancer.

[8] Yoen H., Jang M.J., Yi A., Moon W.K., Chang J.M. (2024). Artificial Intelligence for Breast Cancer Detection on Mammography: Factors Related to Cancer Detection. Acad. Radiol..

[9] Calisto F.M. (2024). Human-Centered Design of Personalized Intelligent Agents in Medical Imaging Diagnosis. Ph.D. Thesis.

[10] Calisto F.M., Nunes N., Nascimento J.C. (2022). Modeling Adoption of Intelligent Agents in Medical Imaging. Int. J. Hum.-Comput. Stud..

[11] Cserni G. (2020). Histological Type and Typing of Breast Carcinomas and the WHO Classification Changes over Time. Pathol.—J. Ital. Soc. Anat. Pathol. Diagn. Cytopathol..

[12] Mouabbi J.A., Hassan A., Lim B., Hortobagyi G.N., Tripathy D., Layman R.M. (2022). Invasive Lobular Carcinoma: An Understudied Emergent Subtype of Breast Cancer. Breast Cancer Res. Treat..

[13] Wen X., Yu Y., Yu X., Cheng W., Wang Z., Liu L., Zhang L., Qin L., Tian J. (2019). Correlations between Ultrasonographic Findings of Invasive Lobular Carcinoma of the Breast and Intrinsic Subtypes. Ultraschall Med.-Eur..

[14] McCart Reed A.E., Kalinowski L., Simpson P.T., Lakhani S.R. (2021). Invasive Lobular Carcinoma of the Breast: The Increasing Importance of This Special Subtype. Breast Cancer Res..

[15] Corso G., Fusco N., Guerini-Rocco E., Leonardi M.C., Criscitiello C., Zagami P., Nicolò E., Mazzarol G., La Vecchia C., Pesapane F. (2024). Invasive Lobular Breast Cancer: Focus on Prevention, Genetics, Diagnosis, and Treatment. Semin. Oncol..

[16] Rakha E.A., Tse G.M., Quinn C.M. (2023). An Update on the Pathological Classification of Breast Cancer. Histopathology.

[17] Manning P., Fazeli S., Lim V., Ladd W.A., Eghtedari M., Chong A., Rakow-Penner R., Ojeda-Fournier H. (2022). Invasive Lobular Carcinoma: A Multimodality Imaging Primer. RadioGraphics.

[18] Danzinger S., Pöckl K., Kronawetter G., Pfeifer C., Behrendt S., Gscheidlinger P., Harrasser L., Mühlböck H., Dirschlmayer W., Schauer C. (2023). Axillary Lymph Node Status and Invasive Lobular Breast Cancer. Wien. Klin. Wochenschr..

[19] Johnson K., Sarma D., Hwang E.S. (2015). Lobular Breast Cancer Series: Imaging. Breast Cancer Res. BCR.

[20] Berg W.A., Gutierrez L., NessAiver M.S., Carter W.B., Bhargavan M., Lewis R.S., Ioffe O.B. (2004). Diagnostic Accuracy of Mammography, Clinical Examination, US, and MR Imaging in Preoperative Assessment of Breast Cancer. Radiology.

[21] Savaridas S.L., Bristow G.D., Cox J. (2016). Invasive Lobular Cancer of the Breast: A Pictorial Essay of Imaging Findings on Mammography, Sonography, and Magnetic Resonance Imaging. Can. Assoc. Radiol. J..

[22] Onega T., Abraham L., Miglioretti D.L., Lee C.I., Henderson L.M., Kerlikowske K., Tosteson A.N.A., Weaver D., Sprague B.L., Bowles E.J.A. (2023). Digital Mammography and Digital Breast Tomosynthesis for Detecting Invasive Lobular and Ductal Carcinoma. Breast Cancer Res. Treat..

[23] Romanucci G., Zantedeschi L., Ventriglia A., Mercogliano S., Bisighin M.V., Cugola L., Bricolo P., Rella R., Mandarà M., Benassuti C. (2021). Lobular Breast Cancer Conspicuity on Digital Breast Tomosynthesis Compared to Synthesized 2D Mammography: A Multireader Study. J. Imaging.

[24] Grubstein A., Rapson Y., Morgenstern S., Gadiel I., Haboosheh A., Yerushalmi R., Cohen M. (2016). Invasive Lobular Carcinoma of the Breast: Appearance on Digital Breast Tomosynthesis. Breast Care.

[25] Kim S.H., Cha E.S., Park C.S., Kang B.J., Whang I.Y., Lee A.W., Song B.J., Park J. (2011). Imaging Features of Invasive Lobular Carcinoma: Comparison with Invasive Ductal Carcinoma. Jpn. J. Radiol..

[26] Watermann D.O., Tempfer C., Hefler L.A., Parat C., Stickeler E. (2005). Ultrasound Morphology of Invasive Lobular Breast Cancer Is Different Compared with Other Types of Breast Cancer. Ultrasound Med. Biol..

[27] Selinko V.L., Middleton L.P., Dempsey P.J. (2004). Role of Sonography in Diagnosing and Staging Invasive Lobular Carcinoma. J. Clin. Ultrasound JCU.

[28] Dołęga-Kozierowski B., Lis M., Marszalska-Jacak H., Koziej M., Celer M., Bandyk M., Kasprzak P., Szynglarewicz B., Matkowski R. (2022). Multimodality Imaging in Lobular Breast Cancer: Differences in Mammography, Ultrasound, and MRI in the Assessment of Local Tumor Extent and Correlation with Molecular Characteristics. Front. Oncol..

[29] Mann R.M., Kuhl C.K., Kinkel K., Boetes C. (2008). Breast MRI: Guidelines from the European Society of Breast Imaging. Eur. Radiol..

[30] Qayyum A., Birdwell R.L., Daniel B.L., Nowels K.W., Jeffrey S.S., Agoston T.A., Herfkens R.J. (2002). MR Imaging Features of Infiltrating Lobular Carcinoma of the Breast: Histopathologic Correlation. AJR Am. J. Roentgenol..

[31] Szabó B.K., Aspelin P., Wiberg M.K., Boné B. (2003). Dynamic MR Imaging of the Breast. Analysis of Kinetic and Morphologic Diagnostic Criteria. Acta Radiol..

[32] Amato F., Bicchierai G., Cirone D., Depretto C., Di Naro F., Vanzi E., Scaperrotta G., Bartolotta T.V., Miele V., Nori J. (2019). Preoperative Loco-Regional Staging of Invasive Lobular Carcinoma with Contrast-Enhanced Digital Mammography (CEDM). Radiol. Med..

[33] Patel B.K., Davis J., Ferraro C., Kosiorek H., Hasselbach K., Ocal T., Pockaj B. (2018). Value Added of Preoperative Contrast-Enhanced Digital Mammography in Patients With Invasive Lobular Carcinoma of the Breast. Clin. Breast Cancer.

[34] Lobbes M.B.I., Neeter L.M.F.H., Raat F., Turk K., Wildberger J.E., van Nijnatten T.J.A., Nelemans P.J. (2023). The Performance of Contrast-Enhanced Mammography and Breast MRI in Local Preoperative Staging of Invasive Lobular Breast Cancer. Eur. J. Radiol..

[35] Diab S.G., Clark G.M., Osborne C.K., Libby A., Allred D.C., Elledge R.M. (1999). Tumor Characteristics and Clinical Outcome of Tubular and Mucinous Breast Carcinomas. J. Clin. Oncol. Off. J. Am. Soc. Clin. Oncol..

[36] Rakha E.A., Lee A.H.S., Evans A.J., Menon S., Assad N.Y., Hodi Z., Macmillan D., Blamey R.W., Ellis I.O. (2010). Tubular Carcinoma of the Breast: Further Evidence to Support Its Excellent Prognosis. J. Clin. Oncol. Off. J. Am. Soc. Clin. Oncol..

[37] Wasif N., McCullough A.E., Gray R.J., Pockaj B.A. (2012). Influence of Uncommon Histology on Breast Conservation Therapy for Breast Cancer-Biology Dictates Technique?. J. Surg. Oncol..

[38] Yang M., Bao W., Zhang X., Kang Y., Haffty B., Zhang L. (2017). Short-Term and Long-Term Clinical Outcomes of Uncommon Types of Invasive Breast Cancer. Histopathology.

[39] Zhang W.-W., Wu S.-G., Ling Y.-H., Sun J.-Y., Long Z.-Q., Hua X., Dong Y., Li F.-Y., He Z.-Y., Lin H.-X. (2018). Clinicopathologic Characteristics and Clinical Outcomes of Pure Type and Mixed Type of Tubular Carcinoma of the Breast: A Single-Institution Cohort Study. Cancer Manag. Res..

[40] Min Y., Bae S.Y., Lee H.-C., Lee J.H., Kim M., Kim J., Lee S.K., Kil W.H., Kim S.W., Lee J.E. (2013). Tubular Carcinoma of the Breast: Clinicopathologic Features and Survival Outcome Compared with Ductal Carcinoma in Situ. J. Breast Cancer.

[41] Harvey J.A. (2007). Unusual Breast Cancers: Useful Clues to Expanding the Differential Diagnosis. Radiology.

[42] Sheppard D.G., Whitman G.J., Huynh P.T., Sahin A.A., Fornage B.D., Stelling C.B. (2000). Tubular Carcinoma of the Breast: Mammographic and Sonographic Features. AJR Am. J. Roentgenol..

[43] Caumo F., Romanucci G., Hunter K., Zorzi M., Brunelli S., Macaskill P., Houssami N. (2018). Comparison of Breast Cancers Detected in the Verona Screening Program Following Transition to Digital Breast Tomosynthesis Screening with Cancers Detected at Digital Mammography Screening. Breast Cancer Res. Treat..

[44] Linda A., Zuiani C., Girometti R., Londero V., Machin P., Brondani G., Bazzocchi M. (2010). Unusual Malignant Tumors of the Breast: MRI Features and Pathologic Correlation. Eur. J. Radiol..

[45] Ghai S., Muradali D., Bukhanov K., Kulkarni S. (2005). Nonenhancing Breast Malignancies on MRI: Sonographic and Pathologic Correlation. Am. J. Roentgenol..

[46] Yılmaz R., Bayramoğlu Z., Emirikçi S., Önder S., Salmaslıoğlu A., Dursun M., Acunaş G., Özmen V. (2018). MR Imaging Features of Tubular Carcinoma: Preliminary Experience in Twelve Masses. Eur. J. Breast Health.

[47] Evans A., Sim Y.T., Thomson K., Jordan L., Purdie C., Vinnicombe S.J. (2016). Shear Wave Elastography of Breast Cancer: Sensitivity According to Histological Type in a Large Cohort. Breast.

[48] Di Saverio S., Gutierrez J., Avisar E. (2008). A Retrospective Review with Long Term Follow up of 11,400 Cases of Pure Mucinous Breast Carcinoma. Breast Cancer Res. Treat..

[49] Budzik M.P., Fudalej M.M., Badowska-Kozakiewicz A.M. (2021). Histopathological Analysis of Mucinous Breast Cancer Subtypes and Comparison with Invasive Carcinoma of No Special Type. Sci. Rep..

[50] Limaiem F., Ahmad F. (2023). Mucinous Breast Carcinoma. StatPearls.

[51] Pintican R., Duma M., Chiorean A., Fetica B., Badan M., Bura V., Szep M., Feier D., Dudea S. (2020). Mucinous versus Medullary Breast Carcinoma: Mammography, Ultrasound, and MRI Findings. Clin. Radiol..

[52] Lam W.W.M., Chu W.C.W., Tse G.M., Ma T.K. (2004). Sonographic Appearance of Mucinous Carcinoma of the Breast. AJR Am. J. Roentgenol..

[53] Larribe M., Thomassin-Piana J., Jalaguier-Coudray A. (2014). Breast Cancers with Round Lumps: Correlations between Imaging and Anatomopathology. Diagn. Interv. Imaging.

[54] Chaudhry A.R., El Khoury M., Gotra A., Eslami Z., Omeroglu A., Omeroglu-Altinel G., Chaudhry S.H., Mesurolle B. (2019). Imaging Features of Pure and Mixed Forms of Mucinous Breast Carcinoma with Histopathological Correlation. Br. J. Radiol..

[55] Zhang L., Jia N., Han L., Yang L., Xu W., Chen W. (2015). Comparative Analysis of Imaging and Pathology Features of Mucinous Carcinoma of the Breast. Clin. Breast Cancer.

[56] Caumo F., Zorzi M., Brunelli S., Romanucci G., Rella R., Cugola L., Bricolo P., Fedato C., Montemezzi S., Houssami N. (2018). Digital Breast Tomosynthesis with Synthesized Two-Dimensional Images versus Full-Field Digital Mammography for Population Screening: Outcomes from the Verona Screening Program. Radiology.

[57] Memis A., Ozdemir N., Parildar M., Ustun E.E., Erhan Y. (2000). Mucinous (Colloid) Breast Cancer: Mammographic and US Features with Histologic Correlation. Eur. J. Radiol..

[58] Han H.-J., Kim S.-H., Cha E.-S., Kim H.-S., Kang B.-J., Choi J.-J., Lee J.-H., Lee A.-W. (2010). Imaging Features of Mucinous Breast Carcinoma. Investig. Magn. Reson. Imaging.

[59] Perkins G., Babiera G., Bedrosian I., Gonzalez-Angulo A., Whitman G., Yang W., Strom E., Woodward W., Tereffe W., Yu T. (2009). Mucinous Breast Carcinoma: Occult Multifocality/Multicentricity in a Favorable Disease. Cancer Res..

[60] Bitencourt A.G.V., Graziano L., Osório C.A.B.T., Guatelli C.S., Souza J.A., Mendonça M.H.S., Marques E.F. (2016). MRI Features of Mucinous Cancer of the Breast: Correlation with Pathologic Findings and Other Imaging Methods. AJR Am. J. Roentgenol..

[61] Monzawa S., Yokokawa M., Sakuma T., Takao S., Hirokaga K., Hanioka K., Adachi S. (2009). Mucinous Carcinoma of the Breast: MRI Features of Pure and Mixed Forms with Histopathologic Correlation. AJR Am. J. Roentgenol..

[62] Kulka J., Madaras L., Floris G., Lax S.F. (2022). Papillary Lesions of the Breast. Virchows Arch..

[63] Ting L., Shi Y.Q., Chen T.B. (2023). Mammary Mucinous Cystadenocarcinoma with Long-Term Follow-Up: Molecular Information and Literature Review. Diagn. Pathol..

[64] Jain E., Kumar A., Jain R., Sharma S. (2021). Primary Mucinous Cystadenocarcinoma of the Breast: A Rare Case Report with Review of Literature. Int. J. Surg. Pathol..

[65] Seong M., Ko E.Y., Han B.-K., Cho S.Y., Cho E.Y., Lee S.K., Lee J.E. (2016). Radiologic Findings of Primary Mucinous Cystadenocarcinoma of the Breast: A Report of Two Cases and a Literature Review. J. Breast Cancer.

[66] Vegni F., D’Alessandris N., Santoro A., Angelico G., Scaglione G., Carlino A., Arciuolo D., Valente M., Sfregola S., Natale M. (2023). Primary Mucinous Cystadenocarcinoma of the Breast Intermixed with Pleomorphic Invasive Lobular Carcinoma: The First Report of This Rare Association. J. Pers. Med..

[67] Wang X., Li Y., Zhao P., Jia H., Dong X., Zhang L., Wang C. (2020). Primary Mucinous Cystadenocarcinoma of the Breast: A Clinicopathologic Analysis of One Case and Review of the Literature. Int. J. Clin. Exp. Pathol..

[68] Deng Y., Xue D., Wang X., Xu S., Ao Q., Hu Z., Wang G. (2012). Mucinous Cystadenocarcinoma of the Breast with a Basal-like Immunophenotype. Pathol. Int..

[69] Foulkes W.D., Smith I.E., Reis-Filho J.S. (2010). Triple-Negative Breast Cancer. N. Engl. J. Med..

[70] Park I., Kim J., Kim M., Bae S.Y., Lee S.K., Kil W.H., Lee J.E., Nam S.J. (2013). Comparison of the Characteristics of Medullary Breast Carcinoma and Invasive Ductal Carcinoma. J. Breast Cancer.

[71] Aksoy A., Odabas H., Kaya S., Bozkurt O., Degirmenci M., Topcu T.O., Aytekin A., Arpaci E., Avci N., Pilanci K.N. (2017). Hormone Receptor Status and Survival of Medullary Breast Cancer Patients. Saudi Med. J..

[72] Limaiem F., Mlika M. (2023). Medullary Breast Carcinoma. StatPearls.

[73] Huober J., Gelber S., Goldhirsch A., Coates A.S., Viale G., Öhlschlegel C., Price K.N., Gelber R.D., Regan M.M., Thürlimann B. (2012). Prognosis of Medullary Breast Cancer: Analysis of 13 International Breast Cancer Study Group (IBCSG) Trials. Ann. Oncol. Off. J. Eur. Soc. Med. Oncol..

[74] Matheus V.S., Kestelman F.P., Canella E.D.O., Djahjah M.C.R., Koch H.A. (2008). Medullary Breast Carcinoma: Anatomo-Radiological Correlation. Radiol. Bras..

[75] Yilmaz E., Lebe B., Balci P., Sal S., Canda T. (2002). Comparison of Mammographic and Sonographic Findings in Typical and Atypical Medullary Carcinomas of the Breast. Clin. Radiol..

[76] Wang X., Xu P., Wang Y., Grant E.G. (2011). Contrast-Enhanced Ultrasonographic Findings of Different Histopathologic Types of Breast Cancer. Acta Radiol..

[77] Tominaga J., Hama H., Kimura N., Takahashi S. (2009). MR Imaging of Medullary Carcinoma of the Breast. Eur. J. Radiol..

[78] Akın Y., Uğurlu M.Ü., Kaya H., Arıbal E. (2016). Diagnostic Value of Diffusion-Weighted Imaging and Apparent Diffusion Coefficient Values in the Differentiation of Breast Lesions, Histpathologic Subgroups and Correlatıon with Prognostıc Factors Using 3.0 Tesla MR. J. Breast Health.

[79] Jeong S.J., Lim H.S., Lee J.S., Park M.H., Yoon J.H., Park J.G., Kang H.K. (2012). Medullary Carcinoma of the Breast: MRI Findings. AJR Am. J. Roentgenol..

[80] Demir S., Sezgin G., Sari A.A., Kucukzeybek B.B., Yigit S., Etit D., Yazici A., Kucukzeybek Y. (2021). Clinicopathological Analysis of Invasive Cribriform Carcinoma of the Breast, with Review of the Literature. Ann. Diagn. Pathol..

[81] Mo C.-H., Ackbarkhan Z., Gu Y.-Y., Chen G., Pang Y.-Y., Dang Y.-W., Feng Z.-B. (2017). Invasive Cribriform Carcinoma of the Breast: A Clinicopathological Analysis of 12 Cases with Review of Literature. Int. J. Clin. Exp. Pathol..

[82] Lee Y.J., Choi B.B., Suh K.S. (2015). Invasive Cribriform Carcinoma of the Breast: Mammographic, Sonographic, MRI, and 18 F-FDG PET-CT Features. Acta Radiol..

[83] Balci P., Başara Akin I., Köremezli N., Güray Durak M., Altay C., Gezer N.S., Sevinç A.İ. (2017). Evaluation and Comparison of Radiologic-Pathologic Findings in Invasive Cribriform Carcinoma of the Breast. Turk. J. Med. Sci..

[84] Jagmohan P., Pool F.J., Putti T.C., Wong J. (2013). Papillary Lesions of the Breast: Imaging Findings and Diagnostic Challenges. Diagn. Interv. Radiol. Ank. Turk..

[85] Kestelman F.P., Gomes C.F.A., Fontes F.B., Marchiori E. (2014). Imaging Findings of Papillary Breast Lesions: A Pictorial Review. Clin. Radiol..

[86] Gültekin M.A., Yabul F.Ç., Temur H.O., Sari L., Yilmaz T.F., Toprak H., Yildiz S. (2022). Papillary Lesions of the Breast: Addition of DWI and TIRM Sequences to Routine Breast MRI Could Help in Differentiation Benign from Malignant. Curr. Med. Imaging.

[87] Durur-Subasi I. (2019). DW-MRI of the Breast: A Pictorial Review. Insights Imaging.

[88] Deman F., Punie K., Laenen A., Neven P., Oldenburger E., Smeets A., Nevelsteen I., Van Ongeval C., Baten A., Faes T. (2020). Assessment of Stromal Tumor Infiltrating Lymphocytes and Immunohistochemical Features in Invasive Micropapillary Breast Carcinoma with Long-Term Outcomes. Breast Cancer Res. Treat..

[89] Acs G., Paragh G., Chuang S.-T., Laronga C., Zhang P.J. (2009). The Presence of Micropapillary Features and Retraction Artifact in Core Needle Biopsy Material Predicts Lymph Node Metastasis in Breast Carcinoma. Am. J. Surg. Pathol..

[90] Nangong J., Cheng Z., Yu L., Zheng X., Ding G. (2022). Invasive Micropapillary Breast Carcinoma: A Retrospective Study on the Clinical Imaging Features and Pathologic Findings. Front. Surg..

[91] Adrada B., Arribas E., Gilcrease M., Yang W.T. (2009). Invasive Micropapillary Carcinoma of the Breast: Mammographic, Sonographic, and MRI Features. AJR Am. J. Roentgenol..

[92] Jones K.N., Guimaraes L.S., Reynolds C.A., Ghosh K., Degnim A.C., Glazebrook K.N. (2013). Invasive Micropapillary Carcinoma of the Breast: Imaging Features with Clinical and Pathologic Correlation. AJR Am. J. Roentgenol..

[93] Günhan-Bilgen I., Zekioglu O., Ustün E.E., Memis A., Erhan Y. (2002). Invasive Micropapillary Carcinoma of the Breast: Clinical, Mammographic, and Sonographic Findings with Histopathologic Correlation. AJR Am. J. Roentgenol..

[94] Yun S.U., Choi B.B., Shu K.S., Kim S.M., Seo Y.D., Lee J.S., Chang E.S. (2012). Imaging Findings of Invasive Micropapillary Carcinoma of the Breast. J. Breast Cancer.

[95] Alsharif S., Daghistani R., Kamberoğlu E.A., Omeroglu A., Meterissian S., Mesurolle B. (2014). Mammographic, Sonographic and MR Imaging Features of Invasive Micropapillary Breast Cancer. Eur. J. Radiol..

[96] Kamitani K., Kamitani T., Ono M., Toyoshima S., Mitsuyama S. (2012). Ultrasonographic Findings of Invasive Micropapillary Carcinoma of the Breast: Correlation between Internal Echogenicity and Histological Findings. Breast Cancer.

[97] Wells C.A., El-Ayat G.A. (2007). Non-Operative Breast Pathology: Apocrine Lesions. J. Clin. Pathol..

[98] Vranic S., Gatalica Z. (2022). An Update on the Molecular and Clinical Characteristics of Apocrine Carcinoma of the Breast. Clin. Breast Cancer.

[99] Provenzano E., Gatalica Z., Vranic S. (2019). Carcinoma with Apocrine Differentiation.

[100] D’Arcy C., Quinn C. (2019). Apocrine Lesions of the Breast: Part 1 of a Two-Part Review: Benign, Atypical and in Situ Apocrine Proliferations of the Breast. J. Clin. Pathol..

[101] Lee H., Kang S.W., Lee J.E., Jeong W.G., Lee J.S., Park M.H., Lim H.S. (2022). Malignant Apocrine Lesions of the Breast: Multimodality Imaging Findings and Biologic Features. J. Breast Cancer.

[102] Gilles R., Lesnik A., Guinebretière J.M., Tardivon A., Masselot J., Contesso G., Vanel D. (1994). Apocrine Carcinoma: Clinical and Mammographic Features. Radiology.

[103] Kim J.M., Kim S.Y., Oh M.H., Lee J.E. (2016). A Rare Case of Invasive Apocrine Carcinoma of the Breast with Unusual Radiologic Findings. Iran. J. Radiol..

[104] Seo K.-J., An Y.Y., Whang I.Y., Chang E.D., Kang B.J., Kim S.H., Park C.S., Kim J.S., Hong H. (2015). Sonography of Invasive Apocrine Carcinoma of the Breast in Five Cases. Korean J. Radiol..

[105] Gokalp G., Topal U., Haholu A., Kizilkaya E. (2006). Apocrine Carcinoma of the Breast: Mammography and Ultrasound Findings. Eur. J. Radiol. Extra.

[106] Onoue S., Katoh T., Chigira H., Matsuo K., Suzuki M., Shibata Y., Maeda M. (1997). A Case of Apocrine Carcinoma of the Breast Presenting as Two Cysts. Breast Cancer.

[107] Jia Y., He C., Liu L., Sun L., Chen Y., Luo Y., Yu T. (2019). A Retrospective Study of the Imaging and Pathological Features of Metaplastic Breast Carcinoma and Review of the Literature. Med. Sci. Monit. Int. Med. J. Exp. Clin. Res..

[108] Esbah O., Turkoz F.P., Turker I., Durnali A., Ekinci A.S., Bal O., Sonmez O.U., Budakoglu B., Arslan U.Y., Oksuzoglu B. (2012). Metaplastic Breast Carcinoma: Case Series and Review of the Literature. Asian Pac. J. Cancer Prev. APJCP.

[109] Tse G.M., Tan P.H., Putti T.C., Lui P.C.W., Chaiwun B., Law B.K.B. (2006). Metaplastic Carcinoma of the Breast: A Clinicopathological Review. J. Clin. Pathol..

[110] Toumi Z., Bullen C., Tang A.C.S., Dalal N., Ellenbogen S. (2011). Metaplastic Breast Carcinoma: A Case Report and Systematic Review of the Literature. Pathol. Int..

[111] Wang S., Lou J., Zou Q., Jiang Y., Wang S., Shi H. (2023). Metaplastic Carcinoma of the Breast: MRI Features with Clinical and Histopathologic Correlation. Acad. Radiol..

[112] Pezzi C.M., Patel-Parekh L., Cole K., Franko J., Klimberg V.S., Bland K. (2007). Characteristics and Treatment of Metaplastic Breast Cancer: Analysis of 892 Cases from the National Cancer Data Base. Ann. Surg. Oncol..

[113] Donato H., Candelária I., Oliveira P., Gonçalo M., Caseiro-Alves F. (2018). Imaging Findings of Metaplastic Carcinoma of the Breast with Pathologic Correlation. J. Belg. Soc. Radiol..

[114] Choi B.B., Shu K.S. (2012). Metaplastic Carcinoma of the Breast: Multimodality Imaging and Histopathologic Assessment. Acta Radiol..

[115] Park J.M., Han B.K., Moon W.K., Choe Y.H., Ahn S.H., Gong G. (2000). Metaplastic Carcinoma of the Breast: Mammographic and Sonographic Findings. J. Clin. Ultrasound JCU.

[116] Yang W.T., Hennessy B., Broglio K., Mills C., Sneige N., Davis W.G., Valero V., Hunt K.K., Gilcrease M.Z. (2007). Imaging Differences in Metaplastic and Invasive Ductal Carcinomas of the Breast. AJR Am. J. Roentgenol..

[117] Velasco M., Santamaría G., Ganau S., Farrús B., Zanón G., Romagosa C., Fernández P.L. (2005). MRI of Metaplastic Carcinoma of the Breast. AJR Am. J. Roentgenol..

[118] Wang J., Wei B., Albarracin C.T., Hu J., Abraham S.C., Wu Y. (2014). Invasive Neuroendocrine Carcinoma of the Breast: A Population-Based Study from the Surveillance, Epidemiology and End Results (SEER) Database. BMC Cancer.

[119] Lavigne M., Menet E., Tille J.-C., Lae M., Fuhrmann L., Bonneau C., Deniziaut G., Melaabi S., Ng C.C.K., Marchiò C. (2018). Comprehensive Clinical and Molecular Analyses of Neuroendocrine Carcinomas of the Breast. Mod. Pathol..

[120] Pareja F., D’Alfonso T.M. (2020). Neuroendocrine Neoplasms of the Breast: A Review Focused on the Updated World Health Organization (WHO) 5th Edition Morphologic Classification. Breast J..

[121] Tan P.H., Ellis I., Allison K., Brogi E., Fox S.B., Lakhani S., Lazar A.J., Morris E.A., Sahin A., Salgado R. (2020). The 2019 World Health Organization Classification of Tumours of the Breast. Histopathology.

[122] Park Y.M., Wu Y., Wei W., Yang W.T. (2014). Primary Neuroendocrine Carcinoma of the Breast: Clinical, Imaging, and Histologic Features. AJR Am. J. Roentgenol..

[123] Park S.E., Cho K.R., Song S.E., Woo O.H., Seo B.K., Lee J. (2021). Mammographic, Sonographic, and MRI Features of Primary Neuroendocrine Carcinoma of the Breast: A Case Report. J. Korean Soc. Radiol..

[124] Jeon C.H., Kim S.M., Jang M., Yun B.L., Ahn H.S., Kim S.-W., Kang E., Park S.Y. (2014). Clinical and Radiologic Features of Neuroendocrine Breast Carcinomas. J. Ultrasound Med. Off. J. Am. Inst. Ultrasound Med..

[125] Irshad A., Ackerman S.J., Pope T.L., Moses C.K., Rumboldt T., Panzegrau B. (2008). Rare Breast Lesions: Correlation of Imaging and Histologic Features with WHO Classification. RadioGraphics.

[126] Bergstrom C., Porembka J., Fang Y., Sarode V., Syed S. (2019). Primary Neuroendocrine Carcinoma of the Breast. Breast J..

[127] Richardson E.T., Jo V.Y., Schnitt S.J. (2023). Salivary Gland–like Tumors of the Breast: A Comparison of Clinicopathologic Features and Molecular Pathogenesis with Their Salivary Gland Counterparts. Arch. Pathol. Lab. Med..

[128] Jacob J.D., Hodge C., Franko J., Pezzi C.M., Goldman C.D., Klimberg V.S. (2016). Rare Breast Cancer: 246 Invasive Secretory Carcinomas from the National Cancer Data Base. J. Surg. Oncol..

[129] Hoda R.S., Brogi E., Pareja F., Nanjangud G., Murray M.P., Weigelt B., Reis-Filho J.S., Wen H.Y. (2019). Secretory Carcinoma of the Breast: Clinicopathologic Profile of 14 Cases Emphasising Distant Metastatic Potential. Histopathology.

[130] Li D., Xiao X., Yang W., Shui R., Tu X., Lu H., Shi D. (2012). Secretory Breast Carcinoma: A Clinicopathological and Immunophenotypic Study of 15 Cases with a Review of the Literature. Mod. Pathol..

[131] Horowitz D.P., Sharma C.S., Connolly E., Gidea-Addeo D., Deutsch I. (2012). Secretory Carcinoma of the Breast: Results from the Survival, Epidemiology and End Results Database. Breast.

[132] Amott D.H., Masters R., Moore S. (2006). Secretory Carcinoma of the Breast. Breast J..

[133] Paeng M.H., Choi H.-Y., Sung S.H., Moon B.I., Shim S.S. (2003). Secretory Carcinoma of the Breast. J. Clin. Ultrasound.

[134] Mun S.H., Ko E.Y., Han B.-K., Shin J.H., Kim S.J., Cho E.Y. (2008). Secretory Carcinoma of the Breast: Sonographic Features. J. Ultrasound Med. Off. J. Am. Inst. Ultrasound Med..

[135] Guldogan N., Esen G., Kayadibi Y., Taskin F., Alfatli A.O., Boy F.N.S., Balci P., Bugdayci O., Tokat F., Ozturk T. (2023). Adenoid Cystic Carcinoma of the Breast: Multimodality Imaging Findings and Review of the Literature. Acad. Radiol..

[136] Zhang M., Liu Y., Yang H., Jin F., Zheng A. (2022). Breast Adenoid Cystic Carcinoma: A Report of Seven Cases and Literature Review. BMC Surg..

